# Signaling and actin waves at a glance

**DOI:** 10.1242/jcs.263634

**Published:** 2025-08-22

**Authors:** Tatsat Banerjee, Yu Deng, Dhiman Sankar Pal, Huiwang Zhan, Pablo A. Iglesias, Peter N. Devreotes

**Affiliations:** ^1^ Department of Cell Biology and Center for Cell Dynamics, School of Medicine, Johns Hopkins University, Baltimore, MD 21205, USA; ^2^ Department of Chemical and Biomolecular Engineering, Whiting School of Engineering, Johns Hopkins University, Baltimore, MD 21218, USA; ^3^ Department of Electrical and Computer Engineering, Whiting School of Engineering, Johns Hopkins University, Baltimore, MD 21218, USA; ^4^ Department of Biological Chemistry, School of Medicine, Johns Hopkins University, Baltimore, MD 21205, USA

**Keywords:** Cortical waves, Signal transduction, Cell migration, Biophysical organization, Chemotaxis, Pattern formation

## Abstract

Waves of signaling and cytoskeletal components, which can be easily seen propagating on the ventral surface of a cell, are a systemic feature of biochemical networks that define the spatiotemporal dynamics of diverse cell physiological processes. In this Cell Science at a Glance article and the accompanying poster, we summarize the origin, mathematical basis, and function of signaling and actin waves from systems biology and biophysics perspectives, focusing on cell migration and polarity. We describe how waves control membrane protrusion morphologies, how different proteins and lipids are organized within the waves by distinct mechanisms, and how excitable network-based mathematical models can explain wave patterns and predict cell behavior. We further delineate how specific components interact biochemically to generate these dynamic patterns. Finally, we provide a set of generalizable underlying biophysical principles to describe the exquisite subcellular organization of signaling and cytoskeletal events, membrane symmetry breaking, protein compartmentalization and wave propagation.

## Teaser

**Summary:** A summary of how signal transduction and cytoskeletal components form dynamic wave patterns at the plasma membrane and cortex, dissecting how these define spatiotemporal dimensions for cellular processes.

**Figure:**
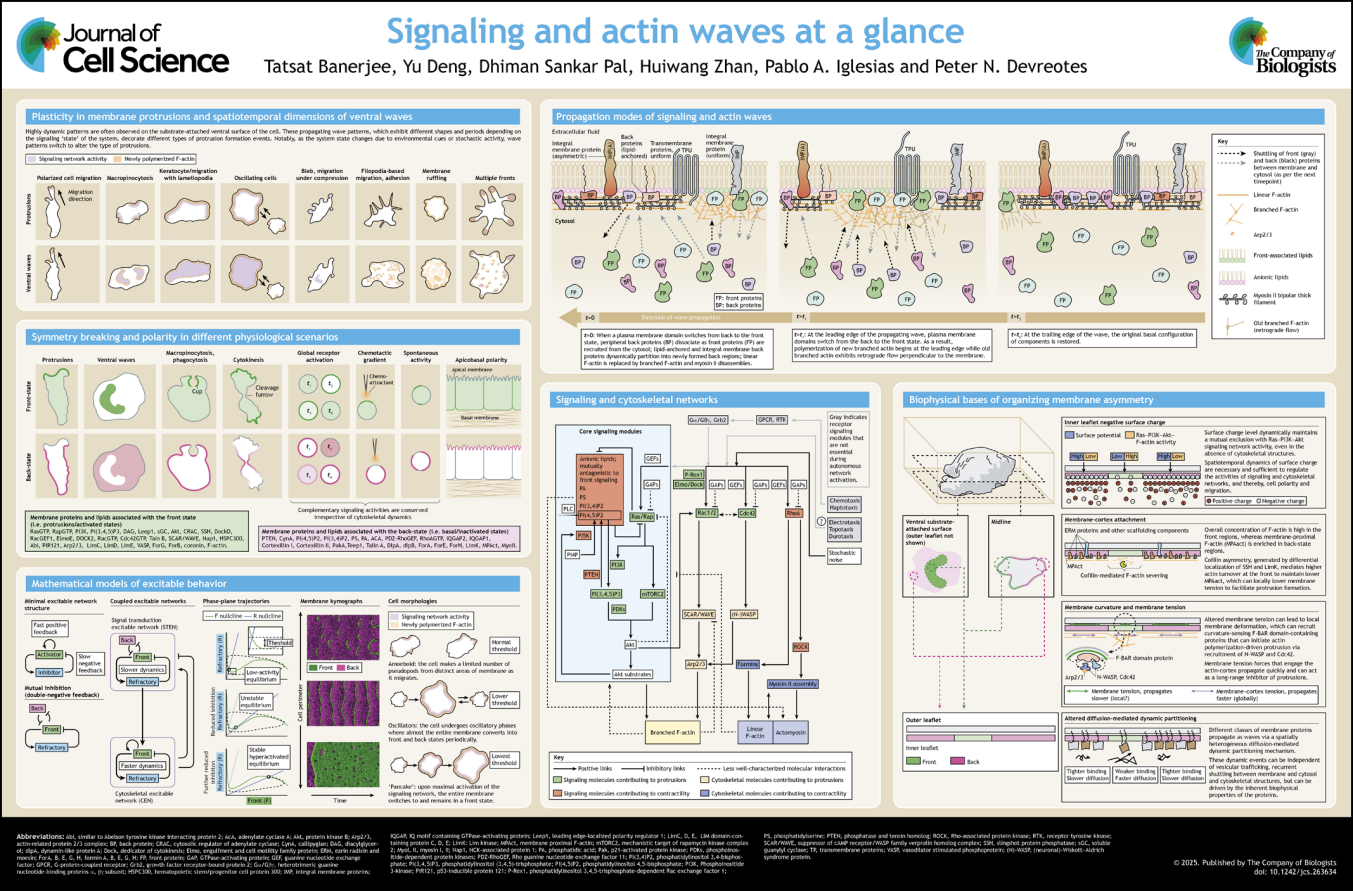
See supplementary information for a high-resolution version of the poster.

## Introduction

Cell signaling networks regulate overall cell physiology, including growth, division, nutrient uptake, energy homeostasis, polarity and migration. Traditionally, signaling cascades were viewed as a series of protein–protein, protein–lipid or lipid–lipid interactions triggered stepwise by surface receptors responding to environmental cues. In reality, these pathways are rarely simple, often featuring complex feedback loops, crosstalk and redundancies. These nonlinearities make cells highly dynamic systems in which numerous components tightly coordinate to define the timing and location of different biological events. These activities shift as needed, guided by adjustments to the strength of key feedback loops, ensuring the cell adapts effectively to changing external conditions. Furthermore, many signaling events are often activated in a receptor-independent fashion where pulsatile, asymmetric activities are simply triggered by stochastic fluctuations within the cell (Sasaki et al., 2007; Miyanaga et al., 2007; Hecht et al., 2010; Huang et al., 2013).

Rhythmicity and dynamic patterns of numerous signaling and cytoskeletal components have been observed in the plasma membrane (PM) and cortex of a wide range of cell types throughout the phylogenetic tree (Box 1). These activities are best visualized as two-dimensional wave patterns that propagate across the substrate-attached ventral surface of the cell. Recent advances in microscopy, genetically encoded biosensors, synthetic biology tools and mathematical modeling approaches have facilitated the detailed deconstruction of the morphological dynamics of these patterns in different physiological scenarios. Notably, these waves not only help cells to organize and sustain their basal activity under stochastic fluctuations but also enable cells to quickly respond to external cues by using them as a template to alter the spatiotemporal activities of numerous components, commensurate with the strength and type of receptor inputs.

Box 1. Physiological roles of actin and signaling waves Since Vicker's discovery of propagating actin ventral waves in *Dictyostelium* (Vicker et al., 1997; Vicker, 2000, 2002), waves and oscillations have been observed across different cells and organisms. Gerisch and colleagues characterized the waves in giant electrofused *Dictyostelium* cells (Bretschneider et al., 2004, 2009; Gerisch et al., 2009, 2011, 2012; Gerhardt et al., 2014; Lange et al., 2016; Ecke et al., 2023; Jasnin et al., 2019). The Ueda and Devreotes laboratories have demonstrated that numerous signaling events, such as PI(3,4,5)P3 production, accompany the waves (Huang et al., 2013; Xiong et al., 2010; Tang et al., 2014; Lin et al., 2024; Deng et al., 2024 preprint; Fukushima et al., 2019; Matsuoka and Ueda, 2018; Arai et al., 2010; Banerjee et al., 2023, 2022). Similar patterns have been observed in neurons and astrocytes (Ruthel and Banker, 1998; Kakumoto and Nakata, 2013; O'Neill et al., 2023), neutrophils (Weiner et al., 2007; Millius et al., 2009), macrophages (Masters et al., 2016), T cells (Lam Hui et al., 2014), mast cells (Wu et al., 2013, 2018), dendritic cells (Stankevicins et al., 2020), endothelial cells (Riedl et al., 2023), keratocytes (Barnhart et al., 2011, 2017), oocytes and embryos (Bement et al., 2015), and various cancer cells (Zhan et al., 2020; Graessl et al., 2017).

Ventral waves regulate cell polarity and random migration in *Dictyostelium*, human neutrophils and several types of cancer cells by controlling protrusion dynamics (Miao et al., 2019, 2017; Devreotes et al., 2017; van Haastert et al., 2017; Banerjee et al., 2022; Weiner et al., 2007; Zhan et al., 2020). These waves also interact with different external cues to define cell polarization (Ecke and Gerisch, 2019; Zhan et al., 2020; Weiner et al., 2007; Bull et al., 2022; Sun et al., 2015; Yang et al., 2023, 2022; Honda et al., 2021; Lange et al., 2016). In fibroblasts and melanoma cells, ventral actin waves mediated by integrin engagement direct sequential assembly and disassembly of focal adhesion components (Case and Waterman, 2011). In *Dictyostelium* and fibroblasts, actin and signaling waves, often in the form of circular dorsal ruffles, generate macropinosomes, whereas diminished wave size dampens macropinocytosis (Kay et al., 2024; Lutton et al., 2023; Veltman et al., 2016; Bernitt et al., 2017; Legg et al., 2007; Saito and Sawai, 2021). Actin and phosphoinositide waves allow macrophages and *Dictyostelium* cells to scan particles to trigger phagocytosis or opt for ‘frustrated’ phagocytosis and not engulf the particle (Gerisch et al., 2009; Banerjee et al., 2022; Masters et al., 2016; Barger et al., 2019). In mammary epithelial cancer cells, increased Ras–PI(3,4,5)P3–actin wave frequency promotes metastasis, possibly by increasing glycolysis and ATP production (Zhan et al., 2025). In *Xenopus laevis* and *Patiria miniata* embryos, excitable waves mediated by Rho activities and delayed negative feedback from actin polymerization regulate the onset of cytokinesis by spatially modulating their propagation zones (Bement et al., 2015, 2024; Michaud et al., 2022, 2021). In mammalian mast cells, concentric or spiral waves of activated Cdc42 and formin-binding protein 17 (FBP17) during metaphase help determine the future cleavage plane during anaphase (Xiao et al., 2017; Tong et al., 2023). In *Dictyostelium*, actin waves drive cytofission (a primitive cell cycle-independent division process) (Flemming et al., 2020), whereas their absence marks the onset of mitosis (Gerisch et al., 2022). During eight-cell-stage mouse embryo morphogenesis, restriction of traveling actin polymerization waves by cell–cell contacts drives the pulsed contraction necessary for lineage specification (Maître et al., 2015). Finally, actin waves, possibly in coordination with microtubule and kinesin dynamics, aid in transporting signaling and cytoskeletal components toward growth cones in neurons, thereby regulating axon development and function (Ruthel and Banker, 1998, 1999; Toriyama et al., 2006; Flynn et al., 2009; Winans et al., 2016).

Waves play crucial roles in directed cell migration, macropinocytosis, phagocytosis, cell cycle regulation, energy production, intracellular transport and more (Box 1). In this Cell Science at a Glance article and the accompanying poster, we primarily focus on the waves and oscillations observed during migration, chemotaxis and macropinocytosis. We first outline how wave patterns define cell protrusions, enabling different migration modes.

Because wave propagation is fundamentally defined by the segregation of membrane states (Banerjee et al., 2022; Arai et al., 2010; Gerisch et al., 2012), we overview the process of symmetry breaking, the initial step whereby membrane components become non-uniformly distributed. We further describe how excitable network-based theoretical models can explain the plasticity of wave propagation modes, introduce how the intertwined intracellular biochemical networks can fit into this framework and illustrate the dynamic molecular events of a propagating wave. Finally, we summarize recent advances in understanding the biophysical principles underlying PM and/or cortex organization. These fundamental principles not only improve our knowledge on symmetry breaking and wave propagation, but also provide a simple, generalizable framework for understanding the intracellular signaling networks that collectively regulate cell polarity, migration and other associated physiological processes.

## Plasticity in membrane protrusions and spatiotemporal dimensions of ventral waves

Migrating cells display a diverse array of protrusions, such as macropinosomes, pseudopodia, lamellipodia, filopodia, blebs and ruffles (Devreotes et al., 2017; SenGupta et al., 2021; Friedl and Alexander, 2011). With some exceptions, these protrusions are consistently decorated by newly polymerized F-actin and by specific signaling molecules, such as the activated form of the small GTPase Ras and phosphatidylinositol (3,4,5)-trisphosphate [PI(3,4,5)P3]. Protrusions are remarkably plastic, and their diversity stems from the overall states of the signaling and cytoskeletal systems in the cells, which are in turn defined by a constellation of gene expression, protein localization and activation kinetics in specific membrane domains, and membrane lipid organization.

Changing the strengths of signaling axes can induce instantaneous transitions in protrusion morphology and cause cells to adopt a new migratory mode (Ladwein and Rottner, 2008; Devreotes et al., 2017). For example, if the level of phosphatidylinositol 4,5-bisphosphate [PI(4,5)P2] in the membrane is lowered or if Ras is activated synthetically, cells of the amoeba *Dictyostelium discoideum* rapidly form large lamellipodia-like structures instead of confined pseudopodia and thereby switch from amoeboid to fast keratocyte- or oscillator-like migratory modes. Conversely, if Ras–phosphoinositide 3-kinase (PI3K) signaling activity is inhibited but cytoskeletal activity is enhanced through recruitment of protein kinase B (PKB or Akt proteins), hereafter referred to as Akt, from the cytosol to membrane, *Dictyostelium* cells make thin, filopodia-like protrusions across the entire membrane (Miao et al., 2019, 2017). Actin and signaling activities are usually tightly correlated but can uncouple under certain mechanochemical conditions. For example, during bleb formation, signaling activities can trigger protrusions without immediate actin polymerization (Weems et al., 2023; Zatulovskiy et al., 2014; Schick and Raz, 2022).

Numerous components of signaling and cytoskeletal networks form propagating wave patterns, most easily observed on the substrate-attached, ventral cell surface. Initially discovered in *Dictyostelium*, these dynamic waves have since been observed in many different cell types and organisms (see Box 1) and they have been shown to underlie and define the dynamics of different protrusions. As in protrusions, signaling and cytoskeletal components mark these ventral waves (also known as ‘cortical waves’). These waves do not significantly deform the cell against the substrate but cause protrusion formation as they reach the cell edge. Combined theoretical and experimental studies have demonstrated that these waves are manifestations of biochemically excitable networks (see section ‘Mathematical models of excitable behavior’ below). Excitable networks are characterized by threshold – a level of activity below which little response is elicited but above which a highly amplified response ensues.

The threshold of the network can be manipulated by altering levels of signaling activities. Synthetically lowering membrane PI(4,5)P2 levels or elevating GTP-bound Ras (RasGTP) activity, for example, increases the speed and propagation range of signaling and actin waves. As they propagate further, these enhanced waves convert confined pseudopodia into wider lamellipodia, as noted above (Miao et al., 2017; Zhan et al., 2020). Many additional perturbations, by changing the activity levels of signaling and/or cytoskeletal components, can alter the threshold of the network and produce correlated changes in wave and protrusion dynamics, indicating that these morphological structures fall on a continuum of different protrusions and corresponding ventral waves (see poster) (Miao et al., 2019; Bhattacharya et al., 2020; Kuhn et al., 2024 preprint; Edwards et al., 2018; Yang et al., 2023; Deng et al., 2024 preprint). The striking correlation strongly suggests that the network threshold determines the size and shape of protrusions and that ventral waves act as a reliable proxy for protrusion dynamics, providing information-rich two-dimensional readouts (Devreotes et al., 2017; Gerhardt et al., 2014; Taniguchi et al., 2013).

## Symmetry breaking and polarity in different physiological scenarios

During different physiological scenarios, the membrane becomes dynamically demarcated into ‘activated’ versus ‘inactivated’ or ‘basal’ state regions (Swaney et al., 2010; Ridley et al., 2003). During polarized cell migration, activated states largely appear at the front of the cell, where the membrane protrudes, whereas inactivated states mostly occupy the back of the cell, where the membrane retracts. Analogously, as ventral waves propagate, the PM–cortex undergoes corresponding segregation into activated and inactivated regions. For simplicity, we will hereafter refer to activities or molecules associated with these state regions as ‘front’ or ‘back’ states (Gerisch et al., 2011; Banerjee et al., 2022, 2023). Numerous signaling and cytoskeletal events, such as the activation of Ras, Rap, Rac and Cdc42 signaling, PI(3,4,5)P3 production and branched actin polymerization, specifically self-organize into front-state regions, whereas the PI(3,4,5)P3 regulator PTEN, activated RhoA–ROCK and assembled myosin II are dynamically depleted from the front-state regions and localize into back-state regions (see poster for a list of components associated with back and front states) (SenGupta et al., 2021; Shellard and Mayor, 2020; Bagorda and Parent, 2008).

This self-organization is preserved across physiological processes, such as macropinocytosis, phagocytosis, cytokinesis and apicobasal polarity generation, in diverse cell types (see poster and Box 1). For example, during cytokinesis, front or back molecules and activities localize to the poles or cleavage furrow, respectively (Janetopoulos and Devreotes, 2006; Brill et al., 2011); similarly, during macropinocytosis (Kay et al., 2024) and phagocytosis (Gerisch et al., 2009; Masters et al., 2016; Banerjee et al., 2022), front or back molecules and events decorate or vacate the ‘cup’-like section of the membrane, respectively. Importantly, the front–back spatiotemporal separation of signaling molecules is typically maintained in the absence of actin polymerization or myosin-driven cytoskeletal activities. In addition, when these spontaneous activities are overridden by global receptor inputs, back-state regions transiently switch to the front state everywhere in the PM–cortex, and the system eventually undergoes adaptation. If the cell experiences an external gradient, front states or back states are created towards or away from the stimulus, respectively (Arai et al., 2010; Parent et al., 1998; Servant et al., 2000; Chua et al., 2024; Sasaki et al., 2004; Wang et al., 2014; Iwamoto et al., 2025; Pipathsouk et al., 2021; Banerjee et al., 2023).

## Propagation modes of signaling and actin waves

Several mechanistically distinct processes facilitate the wave-like propagation of signaling and cytoskeletal molecules on the PM–cortex, yet these processes are tightly coordinated so that different waves can appear, propagate and collapse synchronously while maintaining appropriate time delays between their respective peaks. The concentration of different phospholipids in a particular region of the membrane plays a key role in initiating wave propagation. In back-state regions, high levels of major anionic phospholipids, such as PI(4,5)P2 and phosphatidylserine (PS), are maintained (Banerjee et al., 2022; Gerisch et al., 2011; Fukushima et al., 2019; Masters et al., 2016; Fairn et al., 2009). As the levels of these lipids inside a specific membrane domain decrease, levels of front membrane components and events increase, and the membrane region switches from being the back state to being the front state. During this switch, linear formin-based F-actin structures are predominantly replaced by the branched F-actin cortex, and myosin II disassembles (Litschko et al., 2019; Freeman et al., 2018; Parsons et al., 2010; Li and Gundersen, 2008; Bement et al., 2024).

It is important to note that the actin polymerization waves that propagate parallel to the membrane are not mediated by the lateral translocation of individual filaments, or treadmilling, but by sequential activation and deactivation of the networks (Box 2). Actin polymerization, that is, Arp2/3-based branched nucleation and monomer addition at the barbed end, mostly pushes the membrane outward perpendicularly. These actin waves maintain tight coordination with other front-associated signaling and cytoskeletal molecules (Gerhardt et al., 2014; Banerjee et al., 2022; Gerisch et al., 2012; Lange et al., 2016). As erstwhile back-state membrane regions switch to the front state owing to signaling activities, actin polymerization ‘waves’ propagate across the plane of the membrane, whereas old branched actin starts to exhibit retrograde flow perpendicular to the membrane. Thus, whereas actin filaments elongating against the PM supply the force for membrane protrusions, the parallel propagation of the waves determines the spatiotemporal range of the protrusions (see poster) (Jasnin et al., 2019; Miao et al., 2019). The distinct actin and actomyosin structures in the front versus back states are primarily driven by waves of different signaling proteins and lipids (Kölsch et al., 2008; Devreotes et al., 2017).

Box 2. Structure of actin waves Lattice light-sheet imaging in neutrophil cells has demonstrated that as the ventral waves of the actin-nucleation machinery propagate outwards towards the cell boundary, they form lamellar protrusions, which can interweave to form complex rosettes to collectively regulate the pathfinding of a fast-moving cell (Fritz-Laylin et al., 2017). Although it is evident that actin structures in waves and protrusions share commonalities, how actin filaments are topographically laid out in propagating waves has remained rather poorly understood at the ultrastructural level. A recent *in situ* cryo-electron tomography study has revealed key structural insights (Jasnin et al., 2019), demonstrating that actin waves do not propagate by elongating pre-existing filaments parallel to the PM, but rather by sequential *de novo* nucleation and depolymerization. Consistent with this, at the single-filament level, filament growth is perpendicular to the direction of wave propagation, suggesting the large-scale propagation events are regulated by upstream signaling activities (Jasnin et al., 2019; Miao et al., 2019). A pair of daughter actin filaments originating from a mother filament with opposite polarity did not exhibit any preference towards (or against) the direction of overall wave propagation, further indicating that the self-organization of mother and/or daughter filaments would probably not be sufficient to direct wave propagation. At each location, as the wave propagates, actin polymerization consists of a complex ‘tent’-like network of filaments, instead of a simple dendritic ‘tree’-like structure that lamellipodia are believed to have. As the wave moves forward, Arp2/3 accumulates and creates more daughter filaments that give rise to additional tent-like actin assemblies, with the newer arrays lifting the older ones to the top of the wave. Notably, these elegant ultrastructures empower the cell to precisely reorient actin waves when required and generate force to push the membrane in the correct spatiotemporal direction.

Waves of the other front- and back-associated proteins also propagate via sequential ‘activated–inactivated’ state switching (Miao et al., 2019; Weiner et al., 2007; Bement et al., 2015; Bretschneider et al., 2009; Wu et al., 2018). When a PM domain switches from the back to the front state, back-state proteins dissociate as front-state proteins are simultaneously recruited from the cytosol. At the trailing edge of the wave, the original basal configuration of components is restored. Although the majority of the front- and back-associated proteins display this ‘shuttling’ behavior, different non-peripheral membrane proteins exhibit dynamic wave pattern formation via an altered diffusion-mediated dynamic partitioning mechanism (Banerjee et al., 2023), as discussed below.

## Mathematical models of excitable behavior

To understand the basics of excitable wave propagation, consider a brush fire ignited by a spark that grows as it consumes nearby fuel. The fire exhausts the fuel in its wake, leaving behind a burnt-out refractory region that cannot reignite immediately. This interplay of activation (fuel ignition), inhibition (fuel depletion) and refractory zones, together with the processes that link the activated regions (such as flying sparks), captures the essence of excitable wave propagation mechanisms in biological systems (Hodgkin and Huxley, 1952a,b; FitzHugh, 1961; Nagumo et al., 1962).

In cell motility, the front state acts as the activator, amplifying itself through positive feedback while simultaneously triggering a slower-acting inhibitor and/or refractory element that accumulates over time to quench the activation (Xiong et al., 2010; Weiner et al., 2007). These dynamics prevent runaway activation at that region while allowing activity to diffuse to adjacent regions and initiate new transient activations such that waves propagate across the cell surface. Positive feedback can be achieved through a double-negative feedback loop (Ferrell, 2002), in which the front and back states mutually suppress each other (see poster) (Li et al., 2018; Banerjee et al., 2022; Matsuoka and Ueda, 2018).

Mathematical models of excitable behavior formalize these concepts. Of note, several similar reaction–diffusion models (often in conjunction with level-set or phase-field methods, which allow computation of cellular morphology changes) have greatly aided the study of actin waves and cell motility (Beta et al., 2023; Imoto et al., 2021; Ghabache et al., 2021; Nishikawa et al., 2014; Kockelkoren et al., 2003). In excitable networks, the balance between activation and inhibition determines the stability of the systems and their response to perturbations. Small disturbances, like minor sparks, dissipate quickly if they remain below a critical threshold. However, a perturbation that surpasses this threshold triggers a self-sustaining wave that propagates through diffusion until the inhibitory feedback halts further activation – just as fire spreads until it exhausts its fuel (Bhattacharya and Iglesias, 2019).

Phase-plane analysis allows us to visualize the state of these two-component systems. Here, nullclines represent conditions where there is a balance of the positive and negative regulators of the variables (front state or refractoriness) and hence their respective levels do not change. The intersections of these nullclines define equilibrium points. If the system is near a stable equilibrium, minor fluctuations are quickly corrected. However, nonlinear feedback mechanisms can push the system into an excitable regime, where a sufficiently strong input generates a cycle of activation followed by a refractory period. The spatial extension of these cycles occurs when activator molecules diffuse across the cell membrane, much like embers carried by the wind igniting new patches of fire.

The threshold for excitation, which determines whether a perturbation can trigger a wave, is influenced by the strengths of positive and negative feedback (Biswas et al., 2021; Shi et al., 2013; Abubaker-Sharif et al., 2025 preprint) and can be likened to the moisture content of the brush in a fire-prone landscape. Dry brush ignites easily, akin to a system with strong positive feedback and a lowered excitation threshold. Conversely, damp brush resists ignition, akin to a system with a higher threshold and suppressed spontaneous wave initiation. In cells, altering the strength of feedback loops similarly changes the threshold (see poster). Increased alterations of feedback loop strengths can make the threshold disappear, inducing synchronized oscillatory expansions and contractions (Miao et al., 2017). Further changes can push the system into a stable front state, driving continuous cellular expansion with a ‘pancake’-like morphology and ultimately catastrophic fragmentation (Edwards et al., 2018).

In motile cells, two coupled excitable networks operate – the signal transduction excitable network (STEN) and the cytoskeletal excitable network (CEN) (Huang et al., 2013; van Haastert et al., 2017). The slower STEN modulates long-range signaling and determines overall movement patterns, whereas the faster CEN controls rapid cytoskeletal rearrangements necessary for protrusion and retraction. Feedback between these networks ensures coordinated motion, with STEN waves dictating the locations of actin-driven protrusions and CEN waves reinforcing these structures to maintain persistent movement (Miao et al., 2019; Banerjee et al., 2025 preprint).

## Signaling and cytoskeletal networks

The excitable nature of signaling and cytoskeletal networks stems from faster positive and slower negative feedback loops. However, the corresponding molecular architectures that form these systemic topologies still need to be delineated. The components that organize these dynamic patterning events might vary depending on the cell and organism. Here, we focus on commonalities that drive wave propagation and polarity on the PM–cortex found in both *Dictyostelium* amoebae and human neutrophils. Slightly different biochemical events appear to bring about excitability in mast cells and *Xenopus* oocytes (Michaud et al., 2021; Fung et al., 2024). As mentioned above, receptor inputs or other external cues are not necessary, and stochastic fluctuations can fully trigger local signaling cascades and cytoskeletal rearrangement.

The core signaling module (see poster) contains positive and negative feedback loops that are represented in the mathematical models. Positive feedback is mediated by small GTPases, such as RasGTP (which defines the front state), which form a mutually inhibitory feedback loop with multiple anionic lipids (which defines the back state), primarily PI(4,5)P2, phosphatidylinositol 3,4-bisphosphate [PI(3,4)P2], PS and phosphatidic acid (PA) (see poster) (Banerjee et al., 2022). The levels of the phosphoinositides are regulated by their respective kinases and phosphatases, but how PS levels are dynamically altered in the inner leaflet remains an open question.

Ras activation also initiates signaling cascades that collectively form the slow negative feedback loop – RasGTP activates mechanistic target of rapamycin complex 2 (mTORC2) kinase and PI3K (Cai et al., 2010; Senoo et al., 2019; Pacold et al., 2000). Activated PI3K then produces PI(3,4,5)P3, whereas PTEN catalyzes the reverse dephosphorylation reaction (Iijima and Devreotes, 2002; Chalhoub and Baker, 2009). PI(3,4,5)P3 facilitates the recruitment of Akt from the cytosol to the membrane, where Akt is fully activated through phosphorylation by mTORC2 and by PI(3,4,5)P3-dependent PDK1 (also known as PDPK1) (Kamimura et al., 2008; Kamimura and Devreotes, 2010; Pal et al., 2023a; Hoxhaj and Manning, 2020). Although activated Akt phosphorylates and activates hundreds of different substrates (Manning and Toker, 2017), which of these closes the negative feedback loop to Ras is unknown (Miao et al., 2019).

These interactions comprise a core module conferring biochemical excitability, but to drive spontaneous cell behavior this module must be coupled to additional cytoskeletal components (CEN) (see poster). Activated Akt in turn activates Rac1 and can also activate Cdc42 (possibly via the kinase PAK1) (Kwon et al., 2000; Li et al., 2003), which would then further relay signals to cytoskeletal components. These pathways converge on the activation of the Wiskott–Aldrich syndrome protein (WASP, encoded by *WAS*) and suppressor of cAMP receptor/WASP family verprolin homolog (SCAR/WAVE) complexes (Veltman et al., 2012; Pollitt and Insall, 2009), which activate Arp2/3 (Pollard and Borisy, 2003) to organize branched actin polymerization and promote cell protrusion at the front. Recent evidence suggests that certain formins can also be associated with front-wave activities (Ecke et al., 2020; Chua et al., 2024; Tong et al., 2024). Notably, Rac1 and Cdc42 can also drive cytoskeletal events without directly depending on the Ras–Rap axis (Yan et al., 2012; Wu et al., 2009; Pal et al., 2023b; Yoo et al., 2010; Bell et al., 2021).

Although these events localize to the front, other STEN and CEN components, such as PTEN, organize at the rear. Non-muscle myosin II and formin-based linear F-actin generate contractile forces at the back (Vicente-Manzanares et al., 2009). In *Dictyostelium*, myosin II assembly is modulated by myosin heavy-chain kinases (Bosgraaf and van Haastert, 2006; van Haastert et al., 2021) and by RasGTP localized at the front. In mammalian cells, RhoA–ROCK signaling controls myosin II activity via regulatory light chain phosphorylation, either directly or by inhibiting myosin phosphatase. RhoA also activates mDia formins, facilitating linear actin polymerization (Rose et al., 2005; Campellone and Welch, 2010). Ultimately, myosin II thick filaments enable contraction at the cell rear.

While many signaling pathways maintain asymmetric patterns despite inhibited cytoskeletal dynamics, cytoskeletal networks also modulate upstream signaling through feedback loops under physiological conditions. For example, branched F-actin positively regulates Ras–PI3K signaling, whereas linear F-actin and actomyosin inhibit it (Kuhn et al., 2024 preprint; Pal et al., 2019; Kölsch et al., 2008; Lee et al., 2010; Wang et al., 2002).

## Biophysical bases of organizing membrane asymmetry

Although specific genetic and biochemical interactions among ∼40 different signaling and cytoskeletal components have been documented in migration and polarity, a complete description of all interactions among the hundreds of components involved seems unlikely to emerge soon (Swaney et al., 2010; Belliveau et al., 2023). Some researchers have begun to consider that membrane states likely depend on fundamental biophysical principles (Devreotes, 2019). Significant insights might be gained by understanding the principles that determine the states before trying to delineate every specific interaction. So far, a limited number of biophysical properties have been identified that both closely correlate with the symmetry breaking processes and can produce outsized phenotypic effects if their values are synthetically altered (Banerjee et al., 2022; Kholodenko et al., 2010). Here, we present some examples of biophysical regulation of PM symmetry breaking and polarization (see poster).

### Inner leaflet negative surface potential

The surface potential on the inner leaflet of the PM plays a crucial role in regulating dynamic signal transduction and cytoskeletal events. High levels of the major anionic phospholipids, as mentioned above, generate a highly negatively charged surface at the inner leaflet, which attracts counterions, including proteins with positively charged domains, creating an electrical double layer (Yeung et al., 2006; Ma et al., 2017a; Goldenberg and Steinberg, 2010). This surface potential (McLaughlin, 1989; Eisenberg et al., 2021) is altered during immunological synapse formation (Ma et al., 2017b) and phagocytosis (Yeung et al., 2006, 2008; Fairn et al., 2009).

Recent reports show that the surface charge is highly dynamic: it transiently decreases at cell protrusions or within front-state regions of ventral waves, even in the absence of the actin cytoskeleton. As the level of anionic lipids decreases inside a specific membrane region, the surface potential decreases and front membrane activities increase, so that the membrane region switches from the back to the front state. Furthermore, synthetically lowering the inner leaflet surface potential is sufficient to activate signaling and cytoskeletal networks; conversely, increasing surface potential subverts receptor input-mediated activation (Banerjee et al., 2022). These observations suggest that propagation of surface potential waves (termed ‘action surface potential’) defines the state of the membrane domains and that lowering this surface potential beyond a particular threshold value at a specific membrane domain triggers signaling and cytoskeletal networks there. Although overall surface potential and front signaling mutually inhibit each other, how numerous signaling components collectively define the resting or basal surface potential, or how changes in surface potential might dynamically alter signaling activities across the cell to facilitate different physiological changes remains to be determined.

### Attachment of membranes to the actin cortex

The strength of PM–cortex attachment, typically mediated via ezrin, radixin and moesin (ERM) proteins, is spatially heterogeneous, and weakening it can affect cell protrusions, as seen in bleb formation (Itoh and Tsujita, 2023; Diz-Muñoz et al., 2013; Sheetz, 2001; Welf et al., 2020). Although F-actin concentration is higher at the front of migrating cells, recent evidence suggests that F-actin that is closer to the membrane enriches at the back. This distribution, possibly generated by higher cofilin-mediated severing of actin filaments at the front, might play a key role in regulating long-range polarity (Bisaria et al., 2020; Kuhn et al., 2024 preprint). Protrusion formation might occur more easily at front regions, whereas the strong PM–cortex attachment at the rear precludes protrusions. Additionally, localization of curved endoplasmic reticulum (ER) in the front and sheet-like flattened ER at the back has been demonstrated to result in formation of stable ER–PM contact sites selectively in the back (Gong et al., 2024). Although membrane contacts with the cortex and ER likely contribute to stable polarity, it is uncertain whether these mechanisms play significant roles when symmetry is broken in the absence of cytoskeletal activities and receptor inputs.

### Alteration in membrane curvature and membrane tension

Membrane and/or cortical tension might act as a global inhibitor of signaling activation to limit protrusion formation, especially when diffusion-based inhibition mechanisms are restricted (Houk et al., 2012; Ghisleni and Gauthier, 2024; Sheetz and Dai, 1996). This inhibition is possibly mediated by decreasing recruitment of membrane curvature-sensing molecules, such as F-BAR-domain-containing proteins, and consequently reducing actin polymerization (Wu et al., 2018). How fast membrane tension propagates and the roles that the PM or cortex separately play have remained controversial questions (Shi et al., 2018). When the membrane is pulled separately from the cortex, membrane tension propagates diffusively, possibly by altering lipid packing (Colom et al., 2018), but when the cortex is engaged, as during actin-based protrusion formation or actomyosin-based contraction, membrane tension propagates quickly and increases globally (De Belly et al., 2023). It is not clear how this tension spreads rapidly, but possible mechanisms include long-range membrane and cortical actin flows. Tension, in turn, plays a role in modifying signaling nodes, such as the phospholipase D2 (PLD2)–mTORC2 axis (Saha et al., 2023; Diz-Muñoz et al., 2016), the FBP17–Cdc42–N-WASP (N-WASP is encoded by *WASL*) axis (Wu et al., 2018), and potentially multiple phosphoinositides (Kuhn et al., 2024 preprint).

### Differential diffusion-driven dynamic partitioning

How a myriad of signaling proteins dynamically compartmentalizes inside a particular membrane domain while the membrane undergoes symmetry breaking has long remained a puzzle. Spatiotemporally controlled shuttling is known to play an important role in the polarization of peripheral membrane protein distributions (Matsuoka et al., 2006; Miao et al., 2019; Weiner et al., 2007; Wu and Liu, 2020; Bement et al., 2015; Bretschneider et al., 2009; Wu et al., 2018), but how compartmentalization is facilitated for lipid-anchored and integral membrane proteins was until recently unclear. With much slower shuttling rates, these classes of proteins undergo spatiotemporal rearrangement while remaining attached to the membrane, driven by ‘dynamic partitioning’, in which different membrane proteins continually sense the composition of the inner leaflet domains and alter their diffusion rate accordingly (Banerjee et al., 2023). A difference in diffusion rates in back-state versus front-state regions is sufficient to drive compartmentalization and wave propagation of tightly bound membrane proteins (as shown for lipid-anchored back proteins and asymmetric integral membrane proteins; see poster), irrespective of vesicular trafficking, shuttling rates and supramolecular cytoskeletal structures. Unlike in traditional lipid rafts, molecular crowding or liquid–liquid phase separation, this partitioning mechanism can facilitate spatially large-scale and temporally dynamic compartmentalization. Biochemical factors that regulate the interaction of lipid-anchored or integral membrane proteins with the lipid bilayer to locally alter their diffusion remain to be identified, but a recent study (Honda et al., 2024 preprint) has suggested that the levels of inner leaflet surface charge, PA and sterol in the membrane can play significant roles.

## Concluding remarks

Rhythmic patterns in biological systems have long captivated biologists and mathematicians alike. In 1952, Turing proposed how reaction and diffusion interactions can produce diverse patterns in development (Turing, 1952). Meinhardt and Gierer (Gierer and Meinhardt, 1972, 1974) explicitly defined local self-amplification and long-range inhibition, inspiring numerous studies to identify similar patterns across diverse biomedical processes. Computational scientists have developed reaction-diffusion models to explain and predict pattern formation and cellular polarity, with or without external cues (Beta et al., 2023; Iglesias and Devreotes, 2012). The role of these waves and oscillations beyond polarity and migration remains controversial (Yang and Wu, 2018; Cheong and Levchenko, 2010). However, these patterns are now established to integrate biochemical information, encode templates for organizing specific cellular events and refine spatiotemporal biomolecular dynamics (see Box 1 for examples).

In recent decades, researchers have sought to understand development, physiology and pathologies from a gene-centric view, yet directly linking a gene expression profile to specific cellular or organismal behaviors has proven elusive (Arias, 2023). Organisms often employ strategies beyond genomic reorganization, enabling two genetically similar cells to exhibit drastically different behaviors. For highly temporally dynamic processes like cell migration and polarization, these strategies often rely on spatiotemporal patterning of signaling molecules. These waves or pulses maintain a spatially asymmetric cell state while serving as pre-existing templates of biophysical and biochemical organization, enabling swift responses to external cues. Interestingly, billions of years of evolution have favored the selection of very similar reaction–diffusion waves from bacteria and lower eukaryotes to humans, even though the specific genes involved have often not necessarily been conserved. This suggests that the cellular design principles are potentially more generalizable than particular genetic components or molecules. Hence, to develop a comprehensive mechanistic understanding of cellular processes, we must investigate the higher-order organizing principles governing genetic and biochemical interactions. Our evolving understanding of network architectures and feedback loops that enable small stochastic events to amplify into large-scale oscillations and propagating waves offer an exciting window into these underlying principles.

## Poster

Poster

## Panel 1. Plasticity in membrane protrusions and spatiotemporal dimensions of ventral waves

Panel 1. Plasticity in membrane protrusions and spatiotemporal dimensions of ventral waves

## Panel 2. Symmetry breaking and polarity in different physiological scenarios

Panel 2. Symmetry breaking and polarity in different physiological scenarios

## Panel 3. Propagation modes of signaling and actin waves

Panel 3. Propagation modes of signaling and actin waves

## Panel 4. Mathematical models of excitable behavior

Panel 4. Mathematical models of excitable behavior

## Panel 5. Signaling and cytoskeletal networks

Panel 5. Signaling and cytoskeletal networks

## Panel 6. Biophysical bases of organizing membrane asymmetry

Panel 6. Biophysical bases of organizing membrane asymmetry

## References

[JCS263634C1] Abubaker-Sharif, B., Banerjee, T., Devreotes, P. N. and Iglesias, P. A. (2025). Learning stochastic reaction-diffusion models from limited data using spatiotemporal features. *bioRxiv*, 2024.10.02.616367. 10.1101/2024.10.02.616367

[JCS263634C2] Arai, Y., Shibata, T., Matsuoka, S., Sato, M. J., Yanagida, T. and Ueda, M. (2010). Self-organization of the phosphatidylinositol lipids signaling system for random cell migration. *Proc. Natl. Acad. Sci. USA* 107, 12399-12404. 10.1073/pnas.0908278107 20562345 PMC2901478

[JCS263634C3] Arias, A. M. (2023). *The Master Builder: How the New Science of the Cell is Rewriting the Story of Life*. UK: Hachette.

[JCS263634C4] Bagorda, A. and Parent, C. A. (2008). Eukaryotic chemotaxis at a glance. *J. Cell Sci.* 121, 2621-2624. 10.1242/jcs.018077 18685153 PMC7213762

[JCS263634C5] Banerjee, T., Biswas, D., Pal, D. S., Miao, Y., Iglesias, P. A. and Devreotes, P. N. (2022). Spatiotemporal dynamics of membrane surface charge regulates cell polarity and migration. *Nat. Cell Biol.* 24, 1499-1515. 10.1038/s41556-022-00997-7 36202973 PMC10029748

[JCS263634C6] Banerjee, T., Matsuoka, S., Biswas, D., Miao, Y., Pal, D. S., Kamimura, Y., Ueda, M., Devreotes, P. N. and Iglesias, P. A. (2023). A dynamic partitioning mechanism polarizes membrane protein distribution. *Nat. Commun.* 14, 7909. 10.1038/s41467-023-43615-2 38036511 PMC10689845

[JCS263634C7] Banerjee, P., Kuhn, J. A., Pal, D. S., Deng, Y., Banerjee, T., Devreotes, P. N. and Iglesias, P. A. (2025). Spatial distribution of cytoskeleton-mediated feedback controls cell polarization: a computational study. *bioRxiv*, 2025.04.12.648264. 10.1101/2025.04.12.648264 PMC12539730 41066514

[JCS263634C8] Barger, S. R., Reilly, N. S., Shutova, M. S., Li, Q., Maiuri, P., Heddleston, J. M., Mooseker, M. S., Flavell, R. A., Svitkina, T., Oakes, P. W. et al. (2019). Membrane-cytoskeletal crosstalk mediated by myosin-i regulates adhesion turnover during phagocytosis. *Nat. Commun.* 10, 1249-1241. 10.1038/s41467-019-09104-1 30890704 PMC6425032

[JCS263634C9] Barnhart, E. L., Lee, K. C., Keren, K., Mogilner, A. and Theriot, J. A. (2011). An adhesion-dependent switch between mechanisms that determine motile cell shape. *PLoS Biol.* 9, e1001059. 10.1371/journal.pbio.1001059 21559321 PMC3086868

[JCS263634C10] Barnhart, E. L., Allard, J., Lou, S. S., Theriot, J. A. and Mogilner, A. (2017). Adhesion-dependent wave generation in crawling cells. *Curr. Biol.* 27, 27-38. 10.1016/j.cub.2016.11.011 27939309 PMC5225140

[JCS263634C11] Bell, G. R. R., Rincón, E., Akdoğan, E. and Collins, S. R. (2021). Optogenetic control of receptors reveals distinct roles for actin- and cdc42-dependent negative signals in chemotactic signal processing. *Nat. Commun.* 12, 6148. 10.1038/s41467-021-26371-z 34785668 PMC8595684

[JCS263634C12] Belliveau, N. M., Footer, M. J., Akdoğan, E., Van Loon, A. P., Collins, S. R. and Theriot, J. A. (2023). Whole-genome screens reveal regulators of differentiation state and context-dependent migration in human neutrophils. *Nat. Commun.* 14, 5770. 10.1038/s41467-023-41452-x 37723145 PMC10507112

[JCS263634C13] Bement, W. M., Leda, M., Moe, A. M., Kita, A. M., Larson, M. E., Golding, A. E., Pfeuti, C., Su, K. C., Miller, A. L., Goryachev, A. B. et al. (2015). Activator-inhibitor coupling between rho signalling and actin assembly makes the cell cortex an excitable medium. *Nat. Cell Biol.* 17, 1471-1483. 10.1038/ncb3251 26479320 PMC4849138

[JCS263634C14] Bement, W. M., Goryachev, A. B., Miller, A. L. and Von Dassow, G. (2024). Patterning of the cell cortex by Rho GTPases. *Nat. Rev. Mol. Cell Biol.* 25, 290-308. 10.1038/s41580-023-00682-z 38172611 PMC12706751

[JCS263634C15] Bernitt, E., Döbereiner, H.-G., Gov, N. S. and Yochelis, A. (2017). Fronts and waves of actin polymerization in a bistability-based mechanism of circular dorsal ruffles. *Nat. Commun.* 8, 15863. 10.1038/ncomms15863 28627511 PMC5481797

[JCS263634C16] Beta, C., Edelstein-Keshet, L., Gov, N. and Yochelis, A. (2023). From actin waves to mechanism and back: how theory aids biological understanding. *eLife* 12, e87181. 10.7554/eLife.87181 37428017 PMC10332813

[JCS263634C17] Bhattacharya, S. and Iglesias, P. A. (2019). Controlling excitable wave behaviors through the tuning of three parameters. *Biol. Cybern.* 113, 61-70. 10.1007/s00422-018-0771-0 30056608

[JCS263634C18] Bhattacharya, S., Banerjee, T., Miao, Y., Zhan, H., Devreotes, P. N. and Iglesias, P. A. (2020). Traveling and standing waves mediate pattern formation in cellular protrusions. *Sci. Adv.* 6, eaay7682. 10.1126/sciadv.aay7682 32821814 PMC7413732

[JCS263634C19] Bisaria, A., Hayer, A., Garbett, D., Cohen, D. and Meyer, T. (2020). Membrane-proximal F-actin restricts local membrane protrusions and directs cell migration. *Science* 368, 1205-1210. 10.1126/science.aay7794 32527825 PMC8283920

[JCS263634C20] Biswas, D., Devreotes, P. N. and Iglesias, P. A. (2021). Three-dimensional stochastic simulation of chemoattractant-mediated excitability in cells. *PLoS Comput. Biol.* 17, e1008803. 10.1371/journal.pcbi.1008803 34260581 PMC8330952

[JCS263634C21] Bosgraaf, L. and Van Haastert, P. J. (2006). The regulation of myosin II in Dictyostelium. *Eur. J. Cell Biol.* 85, 969-979. 10.1016/j.ejcb.2006.04.004 16814425

[JCS263634C22] Bretschneider, T., Diez, S., Anderson, K., Heuser, J., Clarke, M., Müller-Taubenberger, A., Hler, J. and Gerisch, G. (2004). Dynamic actin patterns and Arp2/3 assembly at the substrate-attached surface of motile cells. *Curr. Biol.* 14, 1-10. 10.1016/j.cub.2003.12.005 14711408

[JCS263634C23] Bretschneider, T., Anderson, K., Ecke, M., Müller-Taubenberger, A., Schroth-Diez, B., Ishikawa-Ankerhold, H. C. and Gerisch, G. (2009). The three-dimensional dynamics of actin waves, a model of cytoskeletal self-organization. *Biophys. J.* 96, 2888-2900. 10.1016/j.bpj.2008.12.3942 19348770 PMC3325131

[JCS263634C224] Brill, J. A., Wong, R. and Wilde, A. (2011). Phosphoinositide function in cytokinesis. *Curr. Biol.* 21, R930-R934. 10.1016/j.cub.2011.10.001 22115464

[JCS263634C24] Bull, A. L., Campanello, L., Hourwitz, M. J., Yang, Q., Zhao, M., Fourkas, J. T. and Losert, W. (2022). Actin dynamics as a multiscale integrator of cellular guidance cues. *Front. Cell Dev. Biol.* 10, 873567. 10.3389/fcell.2022.873567 35573675 PMC9092214

[JCS263634C25] Cai, H., Das, S., Kamimura, Y., Long, Y., Parent, C. A. and Devreotes, P. N. (2010). Ras-mediated activation of the torc2-pkb pathway is critical for chemotaxis. *J. Cell. Biol.* 190, 233-245. 10.1083/jcb.201001129 20660630 PMC2930282

[JCS263634C26] Campellone, K. G. and Welch, M. D. (2010). A nucleator arms race: cellular control of actin assembly. *Nat. Rev. Mol. Cell Biol.* 11, 237-251. 10.1038/nrm2867 20237478 PMC2929822

[JCS263634C27] Case, L. B. and Waterman, C. M. (2011). Adhesive F-actin waves: a novel integrin-mediated adhesion complex coupled to ventral actin polymerization. *PLoS ONE* 6, e26631. 10.1371/journal.pone.0026631 22069459 PMC3206032

[JCS263634C28] Chalhoub, N. and Baker, S. J. (2009). PTEN and the PI3-kinase pathway in cancer. *Annu. Rev. Pathol.* 4, 127-150. 10.1146/annurev.pathol.4.110807.092311 18767981 PMC2710138

[JCS263634C29] Cheong, R. and Levchenko, A. (2010). Oscillatory signaling processes: the how, the why and the where. *Curr. Opin. Genet. Dev.* 20, 665-669. 10.1016/j.gde.2010.08.007 20971631 PMC3895451

[JCS263634C30] Chua, X. L., Tong, C. S., Su, M., Xŭ, X. J., Xiao, S., Wu, X. and Wu, M. (2024). Competition and synergy of arp2/3 and formins in nucleating actin waves. *Cell Rep.* 43, 114423. 10.1016/j.celrep.2024.114423 38968072 PMC11378572

[JCS263634C31] Colom, A., Derivery, E., Soleimanpour, S., Tomba, C., Molin, M. D., Sakai, N., González-Gaitán, M., Matile, S. and Roux, A. (2018). A fluorescent membrane tension probe. *Nat. Chem.* 10, 1118-1125. 10.1038/s41557-018-0127-3 30150727 PMC6197433

[JCS263634C32] De Belly, H., Yan, S., Borja Da Rocha, H., Ichbiah, S., Town, J. P., Zager, P. J., Estrada, D. C., Meyer, K., Turlier, H., Bustamante, C. et al. (2023). Cell protrusions and contractions generate long-range membrane tension propagation. *Cell* 186, 3049-3061.e15. 10.1016/j.cell.2023.05.014 37311454 PMC10330871

[JCS263634C33] Deng, Y., Banerjee, T., Pal, D. S., Banerjee, P., Zhan, H., Borleis, J., Igleias, P. A. and Devreotes, P. N. (2024). PIP5K-Ras bistability initiates plasma membrane symmetry breaking to regulate cell polarity and migration. *bioRxiv*, 2024.09.15.613115. 10.1101/2024.09.15.613115

[JCS263634C34] Devreotes, P. (2019). Moving toward molecular mechanisms for chemotaxis in eukaryotic cells. *Mol. Biol. Cell* 30, 2873-2877. 10.1091/mbc.E19-07-0393 31671039 PMC6822590

[JCS263634C35] Devreotes, P. N., Bhattacharya, S., Edwards, M., Iglesias, P. A., Lampert, T. and Miao, Y. (2017). Excitable signal transduction networks in directed cell migration. *Annu. Rev. Cell Dev. Biol.* 33, 103-125. 10.1146/annurev-cellbio-100616-060739 28793794 PMC5792054

[JCS263634C36] Diz-Muñoz, A., Fletcher, D. A. and Weiner, O. D. (2013). Use the force: membrane tension as an organizer of cell shape and motility. *Trends Cell Biol.* 23, 47-53. 10.1016/j.tcb.2012.09.006 23122885 PMC3558607

[JCS263634C37] Diz-Muñoz, A., Thurley, K., Chintamen, S., Altschuler, S. J., Wu, L. F., Fletcher, D. A. and Weiner, O. D. (2016). Membrane tension acts through PLD2 and mTORC2 to limit actin network assembly during neutrophil migration. *PLoS Biol.* 14, e1002474. 10.1371/journal.pbio.1002474 27280401 PMC4900667

[JCS263634C38] Ecke, M. and Gerisch, G. (2019). Co-existence of Ras activation in a chemotactic signal transduction pathway and in an autonomous wave - forming system. *Small GTPases* 10, 72-80. 10.1080/21541248.2016.1268666 28136018 PMC6343538

[JCS263634C39] Ecke, M., Prassler, J., Tanribil, P., Müller-Taubenberger, A., Körber, S., Faix, J. and Gerisch, G. (2020). Formins specify membrane patterns generated by propagating actin waves. *Mol. Biol. Cell* 31, 373-385. 10.1091/mbc.E19-08-0460 31940262 PMC7183788

[JCS263634C40] Ecke, M., Prassler, J. and Gerisch, G. (2023). Fluctuations of formin binding in the generation of membrane patterns. *Biophys. J.* 122, 3386-3394. 10.1016/j.bpj.2023.07.014 37488927 PMC10465725

[JCS263634C41] Edwards, M., Cai, H., Abubaker-Sharif, B., Long, Y., Lampert, T. J. and Devreotes, P. N. (2018). Insight from the maximal activation of the signal transduction excitable network in *Dictyostelium discoideum*. *Proc. Natl. Acad. Sci. USA* 115, E3722-E3730. 10.1073/pnas.1710480115 29602807 PMC5910810

[JCS263634C42] Eisenberg, S., Haimov, E., Walpole, G. F. W., Plumb, J., Kozlov, M. M. and Grinstein, S. (2021). Mapping the electrostatic profiles of cellular membranes. *Mol. Biol. Cell* 32, 301-310. 10.1091/mbc.E19-08-0436 33263429 PMC8098824

[JCS263634C43] Fairn, G. D., Ogata, K., Botelho, R. J., Stahl, P. D., Anderson, R. A., De Camilli, P., Meyer, T., Wodak, S. and Grinstein, S. (2009). An electrostatic switch displaces phosphatidylinositol phosphate kinases from the membrane during phagocytosis. *J. Cell Biol.* 187, 701-714. 10.1083/jcb.200909025 19951917 PMC2806594

[JCS263634C44] Ferrell, J. E. (2002). Self-perpetuating states in signal transduction: positive feedback, double-negative feedback and bistability. *Curr. Opin. Cell. Biol.* 14, 140-148. 10.1016/S0955-0674(02)00314-9 11891111

[JCS263634C45] FitzHugh, R. (1961). Impulses and physiological states in theoretical models of nerve membrane. *Biophys. J.* 1, 445-466. 10.1016/S0006-3495(61)86902-6 19431309 PMC1366333

[JCS263634C46] Flemming, S., Font, F., Alonso, S. and Beta, C. (2020). How cortical waves drive fission of motile cells. *Proc. Natl. Acad. Sci. USA* 117, 6330-6338. 10.1073/pnas.1912428117 32161132 PMC7104017

[JCS263634C47] Flynn, K. C., Pak, C. W., Shaw, A. E., Bradke, F. and Bamburg, J. R. (2009). Growth cone-like waves transport actin and promote axonogenesis and neurite branching. *Dev. Neurobiol.* 69, 761-779. 10.1002/dneu.20734 19513994 PMC2845293

[JCS263634C48] Freeman, S. A., Vega, A., Riedl, M., Collins, R. F., Ostrowski, P. P., Woods, E. C., Bertozzi, C. R., Tammi, M. I., Lidke, D. S., Johnson, P. et al. (2018). Transmembrane pickets connect cyto- and pericellular skeletons forming barriers to receptor engagement. *Cell* 172, 305-317.e10. 10.1016/j.cell.2017.12.023 29328918 PMC5929997

[JCS263634C49] Friedl, P. and Alexander, S. (2011). Cancer invasion and the microenvironment: plasticity and reciprocity. *Cell* 147, 992-1009. 10.1016/j.cell.2011.11.016 22118458

[JCS263634C50] Fritz-Laylin, L. K., Riel-Mehan, M., Chen, B.-C., Lord, S. J., Goddard, T. D., Ferrin, T. E., Nicholson-Dykstra, S. M., Higgs, H., Johnson, G. T., Betzig, E. et al. (2017). Actin-based protrusions of migrating neutrophils are intrinsically lamellar and facilitate direction changes. *eLife* 6, e26990. 10.7554/eLife.26990 28948912 PMC5614560

[JCS263634C51] Fukushima, S., Matsuoka, S. and Ueda, M. (2019). Excitable dynamics of ras triggers spontaneous symmetry breaking of pip3 signaling in motile cells. *J. Cell Sci.* 132, jcs224121. 10.1242/jcs.224121 30745337 PMC6432713

[JCS263634C52] Fung, S. Y. S., Xŭ, X. J. and Wu, M. (2024). Nonlinear dynamics in phosphoinositide metabolism. *Curr. Opin. Cell Biol.* 88, 102373. 10.1016/j.ceb.2024.102373 38797149 PMC11186694

[JCS263634C53] Gerhardt, M., Ecke, M., Walz, M., Stengl, A., Beta, C. and Gerisch, G. (2014). Actin and pip3 waves in giant cells reveal the inherent length scale of an excited state. *J. Cell Sci.* 127, 4507-4517. 10.1242/jcs.156000 25107368

[JCS263634C54] Gerisch, G., Ecke, M., Schroth-Diez, B., Gerwig, S., Engel, U., Maddera, L. and Clarke, M. (2009). Self-organizing actin waves as planar phagocytic cup structures. *Cell Adhes. Migr.* 3, 373-382. 10.4161/cam.3.4.9708 PMC2802751 19855162

[JCS263634C55] Gerisch, G., Ecke, M., Wischnewski, D. and Schroth-Diez, B. (2011). Different modes of state transitions determine pattern in the Phosphatidylinositide-Actin system. *BMC Cell Biol.* 12, 42. 10.1186/1471-2121-12-42 21982379 PMC3199247

[JCS263634C56] Gerisch, G., Schroth-Diez, B., Müller-Taubenberger, A. and Ecke, M. (2012). Pip3 waves and pten dynamics in the emergence of cell polarity. *Biophys. J.* 103, 1170-1178. 10.1016/j.bpj.2012.08.004 22995489 PMC3446687

[JCS263634C57] Gerisch, G., Prassler, J. and Ecke, M. (2022). Patterning of the cell cortex and the localization of cleavage furrows in multi-nucleate cells. *J. Cell Sci.* 135, jcs259648. 10.1242/jcs.259648 35274133 PMC9016623

[JCS263634C58] Ghabache, E., Cao, Y., Miao, Y., Groisman, A., Devreotes, P. N. and Rappel, W. J. (2021). Coupling traction force patterns and actomyosin wave dynamics reveals mechanics of cell motion. *Mol. Syst. Biol.* 17, e10505. 10.15252/msb.202110505 34898015 PMC8666840

[JCS263634C59] Ghisleni, A. and Gauthier, N. C. (2024). Mechanotransduction through membrane tension: it's all about propagation? *Curr. Opin. Cell Biol.* 86, 102294. 10.1016/j.ceb.2023.102294 38101114

[JCS263634C60] Gierer, A. and Meinhardt, H. (1972). A theory of biological pattern formation. *Kybernetik* 12, 30-39. 10.1007/BF00289234 4663624

[JCS263634C61] Goldenberg, N. M. and Steinberg, B. E. (2010). Surface charge: a key determinant of protein localization and function. *Cancer Res.* 70, 1277-1280. 10.1158/0008-5472.CAN-09-2905 20124473

[JCS263634C62] Gong, B., Johnston, J. D., Thiemicke, A., De Marco, A. and Meyer, T. (2024). Endoplasmic reticulum-plasma membrane contact gradients direct cell migration. *Nature* 631, 415-423. 10.1038/s41586-024-07527-5 38867038 PMC11236710

[JCS263634C63] Graessl, M., Koch, J., Calderon, A., Kamps, D., Banerjee, S., Mazel, T., Schulze, N., Jungkurth, J. K., Patwardhan, R., Solouk, D. et al. (2017). An excitable Rho GTPase signaling network generates dynamic subcellular contraction patterns. *J. Cell Biol.* 216, 4271-4285. 10.1083/jcb.201706052 29055010 PMC5716289

[JCS263634C64] Hecht, I., Kessler, D. A. and Levine, H. (2010). Transient localized patterns in noise-driven reaction-diffusion systems. *Phys. Rev. Lett.* 104, 158301. 10.1103/PhysRevLett.104.158301 20482022 PMC2882887

[JCS263634C65] Hodgkin, A. L. and Huxley, A. F. (1952a). Propagation of electrical signals along giant nerve fibers. *Proc. R. Soc. Lond. Ser. B Biol. Sci.* 140, 177-183. 10.1098/rspb.1952.0054 13003922

[JCS263634C66] Hodgkin, A. L. and Huxley, A. F. (1952b). A quantitative description of membrane current and its application to conduction and excitation in nerve. *J. Physiol.* 117, 500-544. 10.1113/jphysiol.1952.sp004764 12991237 PMC1392413

[JCS263634C67] Honda, G., Saito, N., Fujimori, T., Hashimura, H., Nakamura, M. J., Nakajima, A. and Sawai, S. (2021). Microtopographical guidance of macropinocytic signaling patches. *Proc. Natl. Acad. Sci. USA* 118, e2110281118. 10.1073/pnas.2110281118 34876521 PMC8685668

[JCS263634C68] Honda, G., Tanaka, C., Sawai, S. and Yanagisawa, M. (2024). Slow diffusion and signal amplification on membranes regulated by phospholipase D. *bioRxiv*, 2024.07.08.602473. 10.1101/2024.07.08.602473

[JCS263634C69] Houk, A. R., Jilkine, A., Mejean, C. O., Boltyanskiy, R., Dufresne, E. R., Angenent, S. B., Altschuler, S. J., Wu, L. F. and Weiner, O. D. (2012). Membrane tension maintains cell polarity by confining signals to the leading edge during neutrophil migration. *Cell* 148, 175-188. 10.1016/j.cell.2011.10.050 22265410 PMC3308728

[JCS263634C70] Hoxhaj, G. and Manning, B. D. (2020). The PI3K-AKT network at the interface of oncogenic signalling and cancer metabolism. *Nat. Rev. Cancer* 20, 74-88. 10.1038/s41568-019-0216-7 31686003 PMC7314312

[JCS263634C71] Huang, C. H., Tang, M., Shi, C., Iglesias, P. A. and Devreotes, P. N. (2013). An excitable signal integrator couples to an idling cytoskeletal oscillator to drive cell migration. *Nat. Cell Biol.* 15, 1307-1316. 10.1038/ncb2859 24142103 PMC3838899

[JCS263634C72] Iglesias, P. A. and Devreotes, P. N. (2012). Biased excitable networks: how cells direct motion in response to gradients. *Curr. Opin. Cell Biol.* 24, 245-253. 10.1016/j.ceb.2011.11.009 22154943 PMC3415256

[JCS263634C73] Iijima, M. and Devreotes, P. (2002). Tumor suppressor pten mediates sensing of chemoattractant gradients. *Cell* 109, 599-610. 10.1016/S0092-8674(02)00745-6 12062103

[JCS263634C74] Imoto, D., Saito, N., Nakajima, A., Honda, G., Ishida, M., Sugita, T., Ishihara, S., Katagiri, K., Okimura, C., Iwadate, Y. et al. (2021). Comparative mapping of crawling-cell morphodynamics in deep learning-based feature space. *PLoS Comput. Biol.* 17, e1009237. 10.1371/journal.pcbi.1009237 34383753 PMC8360578

[JCS263634C75] Itoh, T. and Tsujita, K. (2023). Exploring membrane mechanics: the role of membrane-cortex attachment in cell dynamics. *Curr. Opin. Cell Biol.* 81, 102173. 10.1016/j.ceb.2023.102173 37224683

[JCS263634C76] Iwamoto, K., Matsuoka, S. and Ueda, M. (2025). Excitable ras dynamics-based screens reveal rasgefx is required for macropinocytosis and random cell migration. *Nat. Commun.* 16, 117. 10.1038/s41467-024-55389-2 39746985 PMC11696275

[JCS263634C77] Janetopoulos, C. and Devreotes, P. (2006). Phosphoinositide signaling plays a key role in cytokinesis. *J. Cell Biol..* 174, 485-490. 10.1083/jcb.200603156 16908667 PMC2064254

[JCS263634C78] Jasnin, M., Beck, F., Ecke, M., Fukuda, Y., Martinez-Sanchez, A., Baumeister, W. and Gerisch, G. (2019). The architecture of traveling actin waves revealed by cryo-electron tomography. *Structure* 27, 1211-1223.e5. 10.1016/j.str.2019.05.009 31230946

[JCS263634C79] Kakumoto, T. and Nakata, T. (2013). Optogenetic control of PIP3: PIP3 is sufficient to induce the actin-based active part of growth cones and is regulated via endocytosis. *PLoS ONE* 8, e70861. 10.1371/journal.pone.0070861 23951027 PMC3737352

[JCS263634C80] Kamimura, Y. and Devreotes, P. N. (2010). Phosphoinositide-dependent protein kinase (pdk) activity regulates phosphatidylinositol 3,4,5-trisphosphate-dependent and -independent protein kinase b activation and chemotaxis. *J. Biol. Chem.* 285, 7938-7946. 10.1074/jbc.M109.089235 20075071 PMC2832944

[JCS263634C81] Kamimura, Y., Xiong, Y., Iglesias, P. A., Hoeller, O., Bolourani, P. and Devreotes, P. N. (2008). Pip3-independent activation of torc2 and pkb at the cell's leading edge mediates chemotaxis. *Curr. Biol.* 18, 1034-1043. 10.1016/j.cub.2008.06.068 18635356 PMC4018231

[JCS263634C82] Kay, R. R., Lutton, J. E., King, J. S. and Bretschneider, T. (2024). Making cups and rings: the ‘stalled-wave’ model for macropinocytosis. *Biochem. Soc. Trans.* 52, 1785-1794. 10.1042/BST20231426 38934501 PMC7616836

[JCS263634C83] Kholodenko, B. N., Hancock, J. F. and Kolch, W. (2010). Signalling ballet in space and time. *Nat. Rev. Mol. Cell Biol.* 11, 414-426. 10.1038/nrm2901 20495582 PMC2977972

[JCS263634C84] Kockelkoren, J., Levine, H. and Rappel, W. J. (2003). Computational approach for modeling intra- and extracellular dynamics. *Phys. Rev. E* 68, 037702. 10.1103/PhysRevE.68.037702 14524934

[JCS263634C85] Kölsch, V., Charest, P. G. and Firtel, R. A. (2008). The regulation of cell motility and chemotaxis by phospholipid signaling. *J. Cell Sci.* 121, 551-559. 10.1242/jcs.023333 18287584 PMC2671295

[JCS263634C86] Kuhn, J., Banerjee, P., Haye, A., Robinson, D. N., Iglesias, P. A. and Devreotes, P. N. (2024). Complementary cytoskeletal feedback loops control signal transduction excitability and cell polarity. *bioRxiv*, 2024.02.13.580131. 10.1101/2024.02.13.580131 PMC12344061 40796751

[JCS263634C87] Kwon, T., Kwon, D. Y., Chun, J., Kim, J. H. and Kang, S. S. (2000). Akt protein kinase inhibits Rac1-GTP binding through phosphorylation at serine 71 of Rac1. *J. Biol. Chem.* 275, 423-428. 10.1074/jbc.275.1.423 10617634

[JCS263634C88] Ladwein, M. and Rottner, K. (2008). On the rho'd: the regulation of membrane protrusions by rho-gtpases. *FEBS Lett.* 582, 2066-2074. 10.1016/j.febslet.2008.04.033 18442478

[JCS263634C89] Lam Hui, K., Kwak, S. I. and Upadhyaya, A. (2014). Adhesion-dependent modulation of actin dynamics in Jurkat T cells. *Cytoskeleton (Hoboken)* 71, 119-135. 10.1002/cm.21156 24382832

[JCS263634C90] Lange, M., Prassler, J., Ecke, M., Müller-Taubenberger, A. and Gerisch, G. (2016). Local ras activation, pten pattern, and global actin flow in the chemotactic responses of oversized cells. *J. Cell Sci.* 129, 3462-3472. 10.1242/jcs.191148 27505897

[JCS263634C91] Lee, S., Shen, Z., Robinson, D. N., Briggs, S. and Firtel, R. A. (2010). Involvement of the cytoskeleton in controlling leading-edge function during chemotaxis. *Mol. Biol. Cell* 21, 1810-1824. 10.1091/mbc.e10-01-0009 20375144 PMC2877640

[JCS263634C92] Legg, J. A., Bompard, G., Dawson, J., Morris, H. L., Andrew, N., Cooper, L., Johnston, S. A., Tramountanis, G. and Machesky, L. M. (2007). N-WASP involvement in dorsal ruffle formation in mouse embryonic fibroblasts. *Mol. Biol. Cell* 18, 678-687. 10.1091/mbc.e06-06-0569 17182853 PMC1783773

[JCS263634C93] Li, R. and Gundersen, G. G. (2008). Beyond polymer polarity: how the cytoskeleton builds a polarized cell. *Nat. Rev. Mol. Cell Biol.* 9, 860-873. 10.1038/nrm2522 18946475

[JCS263634C94] Li, Z., Hannigan, M., Mo, Z., Liu, B., Lu, W., Wu, Y., Smrcka, A. V., Wu, G., Li, L., Liu, M. et al. (2003). Directional sensing requires G beta gamma-mediated PAK1 and PIX alpha-dependent activation of Cdc42. *Cell* 114, 215-227. 10.1016/S0092-8674(03)00559-2 12887923

[JCS263634C95] Li, X., Edwards, M., Swaney, K. F., Singh, N., Bhattacharya, S., Borleis, J., Long, Y., Iglesias, P. A., Chen, J. and Devreotes, P. N. (2018). Mutually inhibitory ras-pi(3,4)p2 feedback loops mediate cell migration. *Proc. Natl. Acad. Sci. USA* 115, E9125-E9134. 10.1073/pnas.1809039115 30194235 PMC6166812

[JCS263634C96] Lin, Y., Pal, D. S., Banerjee, P., Banerjee, T., Qin, G., Deng, Y., Borleis, J., Iglesias, P. A. and Devreotes, P. N. (2024). Ras suppression potentiates rear actomyosin contractility-driven cell polarization and migration. *Nat. Cell Biol.* 26, 1062-1076. 10.1038/s41556-024-01453-4 38951708 PMC11364469

[JCS263634C97] Litschko, C., Brühmann, S., Csiszár, A., Stephan, T., Dimchev, V., Damiano-Guercio, J., Junemann, A., Körber, S., Winterhoff, M., Nordholz, B. et al. (2019). Functional integrity of the contractile actin cortex is safeguarded by multiple diaphanous-related formins. *Proc. Natl. Acad. Sci. USA* 116, 3594-3603. 10.1073/pnas.1821638116 30808751 PMC6397521

[JCS263634C98] Lutton, J. E., Coker, H. L. E., Paschke, P., Munn, C. J., King, J. S., Bretschneider, T. and Kay, R. R. (2023). Formation and closure of macropinocytic cups in Dictyostelium. *Curr. Biol.* 33, 3083-3096.e6. 10.1016/j.cub.2023.06.017 37379843 PMC7614961

[JCS263634C99] Ma, Y., Poole, K., Goyette, J. and Gaus, K. (2017a). Introducing membrane charge and membrane potential to t cell signaling. *Front. Immunol.* 8, 1513. 10.3389/fimmu.2017.01513 29170669 PMC5684113

[JCS263634C100] Ma, Y., Yamamoto, Y., Nicovich, P. R., Goyette, J., Rossy, J., Gooding, J. J. and Gaus, K. (2017b). A FRET sensor enables quantitative measurements of membrane charges in live cells. *Nat. Biotechnol.* 35, 363-370. 10.1038/nbt.3828 28288102

[JCS263634C101] Manning, B. D. and Toker, A. (2017). Akt/pkb signaling: navigating the network. *Cell* 169, 381-405. 10.1016/j.cell.2017.04.001 28431241 PMC5546324

[JCS263634C102] Masters, T. A., Sheetz, M. P. and Gauthier, N. C. (2016). F-actin waves, actin cortex disassembly and focal exocytosis driven by actin-phosphoinositide positive feedback. *Cytoskeleton* 73, 180-196. 10.1002/cm.21287 26915738

[JCS263634C103] Maître, J.-L., Niwayama, R., Turlier, H., Lec, F. and Hiiragi, T. (2015). Pulsatile cell-autonomous contractility drives compaction in the mouse embryo. *Nat. Cell Biol.* 17, 849-855. 10.1038/ncb3185 26075357

[JCS263634C104] Matsuoka, S. and Ueda, M. (2018). Mutual inhibition between pten and pip3 generates bistability for polarity in motile cells. *Nat. Commun.* 9, 4481-4480. 10.1038/s41467-018-06856-0 30367048 PMC6203803

[JCS263634C105] Matsuoka, S., Iijima, M., Watanabe, T. M., Kuwayama, H., Yanagida, T., Devreotes, P. N. and Ueda, M. (2006). Single-molecule analysis of chemoattractant-stimulated membrane recruitment of a ph-domain-containing protein. *J. Cell Sci.* 119, 1071-1079. 10.1242/jcs.02824 16507590

[JCS263634C106] McLaughlin, S. (1989). The electrostatic properties of membranes. *Annu. Rev. Biophys. Biophys. Chem.* 18, 113-136. 10.1146/annurev.bb.18.060189.000553 2660821

[JCS263634C107] Meinhardt, H. and Gierer, A. (1974). Applications of a theory of biological pattern formation based on lateral inhibition. *J. Cell Sci.* 15, 321-346. 10.1242/jcs.15.2.321 4859215

[JCS263634C108] Miao, Y., Bhattacharya, S., Edwards, M., Cai, H., Inoue, T., Iglesias, P. A. and Devreotes, P. N. (2017). Altering the threshold of an excitable signal transduction network changes cell migratory modes. *Nat. Cell Biol.* 19, 329-340. 10.1038/ncb3495 28346441 PMC5394931

[JCS263634C109] Miao, Y., Bhattacharya, S., Banerjee, T., Abubaker-Sharif, B., Long, Y., Inoue, T., Iglesias, P. A. and Devreotes, P. N. (2019). Wave patterns organize cellular protrusions and control cortical dynamics. *Mol. Syst. Biol.* 15, e8585. 10.15252/msb.20188585 30858181 PMC6413885

[JCS263634C110] Michaud, A., Swider, Z. T., Landino, J., Leda, M., Miller, A. L., Von Dassow, G., Goryachev, A. B. and Bement, W. M. (2021). Cortical excitability and cell division. *Curr. Biol.* 31, R553-R559. 10.1016/j.cub.2021.02.053 34033789 PMC8358936

[JCS263634C111] Michaud, A., Leda, M., Swider, Z. T., Kim, S., He, J., Landino, J., Valley, J. R., Huisken, J., Goryachev, A. B., Von Dassow, G. et al. (2022). A versatile cortical pattern-forming circuit based on rho, f-actin, ect2, and rga-3/4. *J. Cell Biol.* 221, e202203017. Epub 2022 Jun 16. 10.1083/jcb.202203017 35708547 PMC9206115

[JCS263634C112] Millius, A., Dandekar, S. N., Houk, A. R. and Weiner, O. D. (2009). Neutrophils establish rapid and robust WAVE complex polarity in an actin-dependent fashion. *Curr. Biol.* 19, 253-259. 10.1016/j.cub.2008.12.044 19200726 PMC2705202

[JCS263634C113] Miyanaga, Y., Matsuoka, S., Yanagida, T. and Ueda, M. (2007). Stochastic signal inputs for chemotactic response in dictyostelium cells revealed by single molecule imaging techniques. *Biosystems* 88, 251-260. 10.1016/j.biosystems.2006.07.011 17184903

[JCS263634C114] Nagumo, J., Arimoto, S. and Yoshizawa, S. (1962). An active pulse transmission line simulating nerve axon. *Proc. IRE.* 50, 2061-2070. 10.1109/JRPROC.1962.288235

[JCS263634C115] Nishikawa, M., Hörning, M., Ueda, M. and Shibata, T. (2014). Excitable signal transduction induces both spontaneous and directional cell asymmetries in the phosphatidylinositol lipid signaling system for eukaryotic chemotaxis. *Biophys. J.* 106, 723-734. 10.1016/j.bpj.2013.12.023 24507613 PMC3944603

[JCS263634C116] O'Neill, K. M., Saracino, E., Barile, B., Mennona, N. J., Mola, M. G., Pathak, S., Posati, T., Zamboni, R., Nicchia, G. P., Benfenati, V. et al. (2023). Decoding natural astrocyte rhythms: dynamic actin waves result from environmental sensing by primary rodent astrocytes. *Adv. Biol. (Weinh)* 7, e2200269. 10.1002/adbi.202200269 36709481

[JCS263634C117] Pacold, M. E., Suire, S., Perisic, O., Lara-Gonzalez, S., Davis, C. T., Walker, E. H., Hawkins, P. T., Stephens, L., Eccleston, J. F. and Williams, R. L. (2000). Crystal structure and functional analysis of ras binding to its effector phosphoinositide 3-kinase gamma. *Cell* 103, 931-943. 10.1016/S0092-8674(00)00196-3 11136978

[JCS263634C118] Pal, D. S., Li, X., Banerjee, T., Miao, Y. and Devreotes, P. N. (2019). The excitable signal transduction networks: movers and shapers of eukaryotic cell migration. *Int. J. Dev. Biol.* 63, 407-416. 10.1387/ijdb.190265pd 31840779 PMC6956983

[JCS263634C119] Pal, D. S., Banerjee, T., Lin, Y., de Trogoff, F., Borleis, J., Iglesias, P. A. and Devreotes, P. N. (2023a). Actuation of single downstream nodes in growth factor network steers immune cell migration. *Dev. Cell* 58, 1170-1188.e7. 10.1016/j.devcel.2023.04.019 37220748 PMC10524337

[JCS263634C120] Pal, D. S., Lin, Y., Zhan, H., Banerjee, T., Kuhn, J., Providence, S. and Devreotes, P. N. (2023b). Optogenetic modulation of guanine nucleotide exchange factors of Ras superfamily proteins directly controls cell shape and movement. *Front. Cell Dev. Biol.* 11, 1195806. 10.3389/fcell.2023.1195806 37492221 PMC10363612

[JCS263634C121] Parent, C. A., Blacklock, B. J., Froehlich, W. M., Murphy, D. B. and Devreotes, P. N. (1998). G protein signaling events are activated at the leading edge of chemotactic cells. *Cell* 95, 81-91. 10.1016/S0092-8674(00)81784-5 9778249

[JCS263634C122] Parsons, J. T., Horwitz, A. R. and Schwartz, M. A. (2010). Cell adhesion: integrating cytoskeletal dynamics and cellular tension. *Nat. Rev. Mol. Cell Biol.* 11, 633-643. 10.1038/nrm2957 20729930 PMC2992881

[JCS263634C123] Pipathsouk, A., Brunetti, R. M., Town, J. P., Graziano, B. R., Breuer, A., Pellett, P. A., Marchuk, K., Tran, N. T., Krummel, M. F., Stamou, D. et al. (2021). The WAVE complex associates with sites of saddle membrane curvature. *J. Cell Biol.* 220, e202003086. 10.1083/jcb.202003086 34096975 PMC8185649

[JCS263634C124] Pollard, T. D. and Borisy, G. G. (2003). Cellular motility driven by assembly and disassembly of actin filaments. *Cell* 112, 453-465. 10.1016/S0092-8674(03)00120-X 12600310

[JCS263634C125] Pollitt, A. Y. and Insall, R. H. (2009). WASP and SCAR/WAVE proteins: the drivers of actin assembly. *J. Cell Sci.* 122, 2575-2578. 10.1242/jcs.023879 19625501 PMC2954249

[JCS263634C126] Ridley, A. J., Schwartz, M. A., Burridge, K., Firtel, R. A., Ginsberg, M. H., Borisy, G., Parsons, J. T. and Horwitz, A. R. (2003). Cell migration: integrating signals from front to back. *Science* 302, 1704-1709. 10.1126/science.1092053 14657486

[JCS263634C127] Riedl, M., Mayer, I., Merrin, J., Sixt, M. and Hof, B. (2023). Synchronization in collectively moving inanimate and living active matter. *Nat. Commun.* 14, 5633. 10.1038/s41467-023-41432-1 37704595 PMC10499792

[JCS263634C128] Rose, R., Weyand, M., Lammers, M., Ishizaki, T., Ahmadian, M. R. and Wittinghofer, A. (2005). Structural and mechanistic insights into the interaction between Rho and mammalian Dia. *Nature* 435, 513-518. 10.1038/nature03604 15864301

[JCS263634C129] Ruthel, G. and Banker, G. (1998). Actin-dependent anterograde movement of growth-cone-like structures along growing hippocampal axons: a novel form of axonal transport? *Cell Motil. Cytoskeleton* 40, 160-173. 10.1002/(SICI)1097-0169(1998)40:2<160::AID-CM5>3.0.CO;2-J 9634213

[JCS263634C130] Ruthel, G. and Banker, G. (1999). Role of moving growth cone-like “wave” structures in the outgrowth of cultured hippocampal axons and dendrites. *J. Neurobiol.* 39, 97-106. 10.1002/(SICI)1097-4695(199904)39:1<97::AID-NEU8>3.0.CO;2-Z 10213456

[JCS263634C131] Saha, S., Town, J. P. and Weiner, O. D. (2023). Mechanosensitive mTORC2 independently coordinates leading and trailing edge polarity programs during neutrophil migration. *Mol. Biol. Cell* 34, ar35. 10.1091/mbc.E22-05-0191 36857159 PMC10162419

[JCS263634C132] Saito, N. and Sawai, S. (2021). Three-dimensional morphodynamic simulations of macropinocytic cups. *iScience* 24, 103087. 10.1016/j.isci.2021.103087 34755081 PMC8560551

[JCS263634C133] Sasaki, A. T., Chun, C., Takeda, K. and Firtel, R. A. (2004). Localized ras signaling at the leading edge regulates pi3k, cell polarity, and directional cell movement. *J. Cell Biol.* 167, 505-518. 10.1083/jcb.200406177 15534002 PMC2172490

[JCS263634C134] Sasaki, A. T., Janetopoulos, C., Lee, S., Charest, P. G., Takeda, K., Sundheimer, L. W., Meili, R., Devreotes, P. N. and Firtel, R. A. (2007). G protein-independent ras/pi3k/f-actin circuit regulates basic cell motility. *J. Cell Biol.* 178, 185-191. 10.1083/jcb.200611138 17635933 PMC2064438

[JCS263634C135] Schick, J. and Raz, E. (2022). Blebs-formation, regulation, positioning, and role in amoeboid cell migration. *Front. Cell Dev. Biol.* 10, 926394. 10.3389/fcell.2022.926394 35912094 PMC9337749

[JCS263634C136] Sengupta, S., Parent, C. A. and Bear, J. E. (2021). The principles of directed cell migration. *Nat. Rev. Mol. Cell Biol.* 22, 529-547. 10.1038/s41580-021-00366-6 33990789 PMC8663916

[JCS263634C137] Senoo, H., Kamimura, Y., Kimura, R., Nakajima, A., Sawai, S., Sesaki, H. and Iijima, M. (2019). Phosphorylated rho-gdp directly activates mtorc2 kinase towards akt through dimerization with ras-gtp to regulate cell migration. *Nat. Cell Biol.* 21, 867-878. 10.1038/s41556-019-0348-8 31263268 PMC6650273

[JCS263634C138] Servant, G., Weiner, O. D., Herzmark, P., Balla, T., Sedat, J. W. and Bourne, H. R. (2000). Polarization of chemoattractant receptor signaling during neutrophil chemotaxis. *Science* 287, 1037-1040. 10.1126/science.287.5455.1037 10669415 PMC2822871

[JCS263634C139] Sheetz, M. P. (2001). Cell control by membrane-cytoskeleton adhesion. *Nat. Rev. Mol. Cell Biol.* 2, 392-396. 10.1038/35073095 11331914

[JCS263634C140] Sheetz, M. P. and Dai, J. (1996). Modulation of membrane dynamics and cell motility by membrane tension. *Trends Cell Biol.* 6, 85-89. 10.1016/0962-8924(96)80993-7 15157483

[JCS263634C141] Shellard, A. and Mayor, R. (2020). All roads lead to directional cell migration. *Trends Cell Biol..* 30, 852-868. 10.1016/j.tcb.2020.08.002 32873438

[JCS263634C142] Shi, C., Huang, C. H., Devreotes, P. N. and Iglesias, P. A. (2013). Interaction of motility, directional sensing, and polarity modules recreates the behaviors of chemotaxing cells. *PLoS Comput. Biol.* 9, e1003122. 10.1371/journal.pcbi.1003122 23861660 PMC3701696

[JCS263634C143] Shi, Z., Graber, Z. T., Baumgart, T., Stone, H. A. and Cohen, A. E. (2018). Cell membranes resist flow. *Cell* 175, 1769-1779.e13. 10.1016/j.cell.2018.09.054 30392960 PMC6541487

[JCS263634C144] Stankevicins, L., Ecker, N., Terriac, E., Maiuri, P., Schoppmeyer, R., Vargas, P., Lennon-Dum'Enil, A. M., Piel, M., Qu, B., Hoth, M. et al. (2020). Deterministic actin waves as generators of cell polarization cues. *Proc. Natl. Acad. Sci. USA* 117, 826-835. 10.1073/pnas.1907845117 31882452 PMC6969493

[JCS263634C145] Sun, X., Driscoll, M. K., Guven, C., Das, S., Parent, C. A., Fourkas, J. T. and Losert, W. (2015). Asymmetric nanotopography biases cytoskeletal dynamics and promotes unidirectional cell guidance. *Proc. Natl. Acad. Sci. USA* 112, 12557-12562. 10.1073/pnas.1502970112 26417076 PMC4611618

[JCS263634C146] Swaney, K. F., Huang, C. H. and Devreotes, P. N. (2010). Eukaryotic chemotaxis: a network of signaling pathways controls motility, directional sensing, and polarity. *Annu. Rev. Biophys.* 39, 265-289. 10.1146/annurev.biophys.093008.131228 20192768 PMC4364543

[JCS263634C147] Tang, M., Wang, M., Shi, C., Iglesias, P. A., Devreotes, P. N. and Huang, C. H. (2014). Evolutionarily conserved coupling of adaptive and excitable networks mediates eukaryotic chemotaxis. *Nat. Commun.* 5, 5175. 10.1038/ncomms6175 25346418 PMC4211273

[JCS263634C148] Taniguchi, D., Ishihara, S., Oonuki, T., Honda-Kitahara, M., Kaneko, K. and Sawai, S. (2013). Phase geometries of two-dimensional excitable waves govern self-organized morphodynamics of amoeboid cells. *Proc. Natl. Acad. Sci. USA* 110, 5016-5021. 10.1073/pnas.1218025110 23479620 PMC3612638

[JCS263634C149] Tong, C. S., Xŭ, X. J. and Wu, M. (2023). Periodicity, mixed-mode oscillations, and multiple timescales in a phosphoinositide-Rho GTPase network. *Cell Rep.* 42, 112857. 10.1016/j.celrep.2023.112857 37494180

[JCS263634C150] Tong, C. S., Su, M., Sun, H., Chua, X. L., Xiong, D., Guo, S., Raj, R., Ong, N. W. P., Lee, A. G., Miao, Y. et al. (2024). Collective dynamics of actin and microtubule and its crosstalk mediated by fhdc1. *Front. Cell Dev. Biol.* 11, 1261117. 10.3389/fcell.2023.1261117 38567385 PMC10985548

[JCS263634C151] Toriyama, M., Shimada, T., Kim, K. B., Mitsuba, M., Nomura, E., Katsuta, K., Sakumura, Y., Roepstorff, P. and Inagaki, N. (2006). Shootin1: a protein involved in the organization of an asymmetric signal for neuronal polarization. *J. Cell Biol.* 175, 147-157. 10.1083/jcb.200604160 17030985 PMC2064506

[JCS263634C152] Turing, A. (1952). The chemical basis of morphogenesis. *Philos. Trans. R. Soc. Lond. B Biol. Sci.* 237, 37-72. 10.1098/rstb.1952.0012 PMC4360114 25750229

[JCS263634C153] van Haastert, P. J., Keizer-Gunnink, I. and Kortholt, A. (2017). Coupled excitable ras and f-actin activation mediates spontaneous pseudopod formation and directed cell movement. *Mol. Biol. Cell.* 28, 922-934. 10.1091/mbc.e16-10-0733 28148648 PMC5385941

[JCS263634C154] van Haastert, P. J. M., Keizer-Gunnink, I., Pots, H., Ortiz-Mateos, C., Veltman, D., Van Egmond, W. and Kortholt, A. (2021). Forty-five years of cGMP research in Dictyostelium: understanding the regulation and function of the cGMP pathway for cell movement and chemotaxis. *Mol. Biol. Cell* 32, ar8. 10.1091/mbc.E21-04-0171 34347507 PMC8684759

[JCS263634C155] Veltman, D. M., King, J. S., Machesky, L. M. and Insall, R. H. (2012). SCAR knockouts in Dictyostelium: WASP assumes SCAR's position and upstream regulators in pseudopods. *J. Cell Biol.* 198, 501-508. 10.1083/jcb.201205058 22891261 PMC3514037

[JCS263634C156] Veltman, D. M., Williams, T. D., Bloomfield, G., Chen, B.-C., Betzig, E., Insall, R. H. and Kay, R. R. (2016). A plasma membrane template for macropinocytic cups. *eLife* 5, 10.7554/eLife.20085. 10.7554/eLife.20085 PMC5154761 27960076

[JCS263634C157] Vicente-Manzanares, M., Ma, X., Adelstein, R. S. and Horwitz, A. R. (2009). Non-muscle myosin II takes centre stage in cell adhesion and migration. *Nat. Rev. Mol. Cell Biol.* 10, 778-790. 10.1038/nrm2786 19851336 PMC2834236

[JCS263634C158] Vicker, M. G. (2000). Reaction-diffusion waves of actin filament polymerization/depolymerization in Dictyostelium pseudopodium extension and cell locomotion. *Biophys. Chem.* 84, 87-98. 10.1016/S0301-4622(99)00146-5 10796025

[JCS263634C159] Vicker, M. G. (2002). Eukaryotic cell locomotion depends on the propagation of self-organized reaction-diffusion waves and oscillations of actin filament assembly. *Exp. Cell Res.* 275, 54-66. 10.1006/excr.2001.5466 11925105

[JCS263634C160] Vicker, M. G., Xiang, W., Plath, P. J. and Wosniok, W. (1997). Pseudopodium extension and amoeboid locomotion in dictyostelium discoideum: Possible autowave behaviour of f-actin. *Physica D* 101, 317-332. 10.1016/S0167-2789(96)00224-2

[JCS263634C161] Wang, F., Herzmark, P., Weiner, O. D., Srinivasan, S., Servant, G. and Bourne, H. R. (2002). Lipid products of PI(3)Ks maintain persistent cell polarity and directed motility in neutrophils. *Nat. Cell Biol.* 4, 513-518. 10.1038/ncb810 12080345

[JCS263634C162] Wang, M. J., Artemenko, Y., Cai, W. J., Iglesias, P. A. and Devreotes, P. N. (2014). The directional response of chemotactic cells depends on a balance between cytoskeletal architecture and the external gradient. *Cell Rep.* 9, 1110-1121. 10.1016/j.celrep.2014.09.047 25437564 PMC4250826

[JCS263634C163] Weems, A. D., Welf, E. S., Driscoll, M. K., Zhou, F. Y., Mazloom-Farsibaf, H., Chang, B. J., Murali, V. S., Gihana, G. M., Weiss, B. G., Chi, J. et al. (2023). Blebs promote cell survival by assembling oncogenic signalling hubs. *Nature* 615, 517-525. 10.1038/s41586-023-05758-6 36859545 PMC10881276

[JCS263634C164] Weiner, O. D., Marganski, W. A., Wu, L. F., Altschuler, S. J. and Kirschner, M. W. (2007). An actin-based wave generator organizes cell motility. *PLoS Biol..* 5, e221. 10.1371/journal.pbio.0050221 17696648 PMC1945041

[JCS263634C165] Welf, E. S., Miles, C. E., Huh, J., Sapoznik, E., Chi, J., Driscoll, M. K., Isogai, T., Noh, J., Weems, A. D., Pohlkamp, T. et al. (2020). Actin-membrane release initiates cell protrusions. *Dev. Cell* 55, 723-736.e8. 10.1016/j.devcel.2020.11.024 33308479 PMC7908823

[JCS263634C166] Winans, A. M., Collins, S. R. and Meyer, T. (2016). Waves of actin and microtubule polymerization drive microtubule-based transport and neurite growth before single axon formation. *eLife* 5, e12387. 10.7554/eLife.12387 26836307 PMC4805541

[JCS263634C167] Wu, M. and Liu, J. (2020). Mechanobiology in cortical waves and oscillations. *Curr. Opin. Cell Biol.* 68, 45-54. 10.1016/j.ceb.2020.08.017 33039945

[JCS263634C168] Wu, Y. I., Frey, D., Lungu, O. I., Jaehrig, A., Schlichting, I., Kuhlman, B. and Hahn, K. M. (2009). A genetically encoded photoactivatable rac controls the motility of living cells. *Nature* 461, 104-108. 10.1038/nature08241 19693014 PMC2766670

[JCS263634C169] Wu, M., Wu, X. and De Camilli, P. (2013). Calcium oscillations-coupled conversion of actin travelling waves to standing oscillations. *Proc. Natl. Acad. Sci. USA* 110, 1339-1344. 10.1073/pnas.1221538110 23297209 PMC3557052

[JCS263634C170] Wu, Z., Su, M., Tong, C., Wu, M. and Liu, J. (2018). Membrane shape-mediated wave propagation of cortical protein dynamics. *Nat. Commun.* 9, 136. 10.1038/s41467-017-02469-1 29321558 PMC5762918

[JCS263634C171] Xiao, S., Tong, C., Yang, Y. and Wu, M. (2017). Mitotic cortical waves predict future division sites by encoding positional and size information. *Dev. Cell* 43, 493-506.e3. 10.1016/j.devcel.2017.10.023 29161593

[JCS263634C172] Xiong, Y., Huang, C. H., Iglesias, P. A. and Devreotes, P. N. (2010). Cells navigate with a local-excitation, global-inhibition-biased excitable network. *Proc. Natl. Acad. Sci. USA* 107, 17079-17086. 10.1073/pnas.1011271107 20864631 PMC2951443

[JCS263634C173] Yan, J., Mihaylov, V., Xu, X., Brzostowski, J. A., Li, H., Liu, L., Veenstra, T. D., Parent, C. A. and Jin, T. (2012). A Gβγ effector, ElmoE, transduces GPCR signaling to the actin network during chemotaxis. *Dev. Cell* 22, 92-103. 10.1016/j.devcel.2011.11.007 22264729 PMC6993146

[JCS263634C174] Yang, Y. and Wu, M. (2018). Rhythmicity and waves in the cortex of single cells. *Philos. Trans. R. Soc. Lond. B Biol. Sci.* 373, 20170116. 10.1098/rstb.2017.0116 29632268 PMC5904302

[JCS263634C175] Yang, Q., Miao, Y., Campanello, L. J., Hourwitz, M. J., Abubaker-Sharif, B., Bull, A. L., Devreotes, P. N., Fourkas, J. T. and Losert, W. (2022). Cortical waves mediate the cellular response to electric fields. *eLife* 11, e73198. 10.7554/eLife.73198.sa2 35318938 PMC8942472

[JCS263634C176] Yang, Q., Miao, Y., Banerjee, P., Hourwitz, M. J., Hu, M., Qing, Q., Iglesias, P. A., Fourkas, J. T., Losert, W. and Devreotes, P. N. (2023). Nanotopography modulates intracellular excitable systems through cytoskeleton actuation. *Proc. Natl. Acad. Sci. USA* 120, e2218906120. 10.1073/pnas.2218906120 37126708 PMC10175780

[JCS263634C177] Yeung, T., Terebiznik, M., Yu, L., Silvius, J., Abidi, W. M., Philips, M., Levine, T., Kapus, A. and Grinstein, S. (2006). Receptor activation alters inner surface potential during phagocytosis. *Science* 313, 347-351. 10.1126/science.1129551 16857939

[JCS263634C178] Yeung, T., Gilbert, G. E., Shi, J., Silvius, J., Kapus, A. and Grinstein, S. (2008). Membrane phosphatidylserine regulates surface charge and protein localization. *Science* 319, 210-213. 10.1126/science.1152066 18187657

[JCS263634C179] Yoo, S. K., Deng, Q., Cavnar, P. J., Wu, Y. I., Hahn, K. M. and Huttenlocher, A. (2010). Differential regulation of protrusion and polarity by pi3k during neutrophil motility in live zebrafish. *Dev. Cell* 18, 226-236. 10.1016/j.devcel.2009.11.015 20159593 PMC2824622

[JCS263634C180] Zatulovskiy, E., Tyson, R., Bretschneider, T. and Kay, R. R. (2014). Bleb-driven chemotaxis of dictyostelium cells. *J. Cell Biol.* 204, 1027-1044. 10.1083/jcb.201306147 24616222 PMC3998804

[JCS263634C181] Zhan, H., Bhattacharya, S., Cai, H., Iglesias, P. A., Huang, C. H. and Devreotes, P. N. (2020). An excitable ras/pi3k/erk signaling network controls migration and oncogenic transformation in epithelial cells. *Dev. Cell* 54, 608-623.e5. 10.1016/j.devcel.2020.08.001 32877650 PMC7505206

[JCS263634C182] Zhan, H., Pal, D. S., Borleis, J., Deng, Y., Long, Y., Janetopoulos, C., Huang, C. H. and Devreotes, P. N. (2025). Self-organizing glycolytic waves tune cellular metabolic states and fuel cancer progression. *Nat. Commun.* 16, 5563. 10.1038/s41467-025-60596-6 40593532 PMC12217304

