# Supplementary figures and images for: Signaling and actin waves at a glance
Source: J Cell Sci. 2025 Aug 22;138(16):jcs263634. doi: 10.1242/jcs.263634 (PMC12380181; doi:10.1242/jcs.263634)

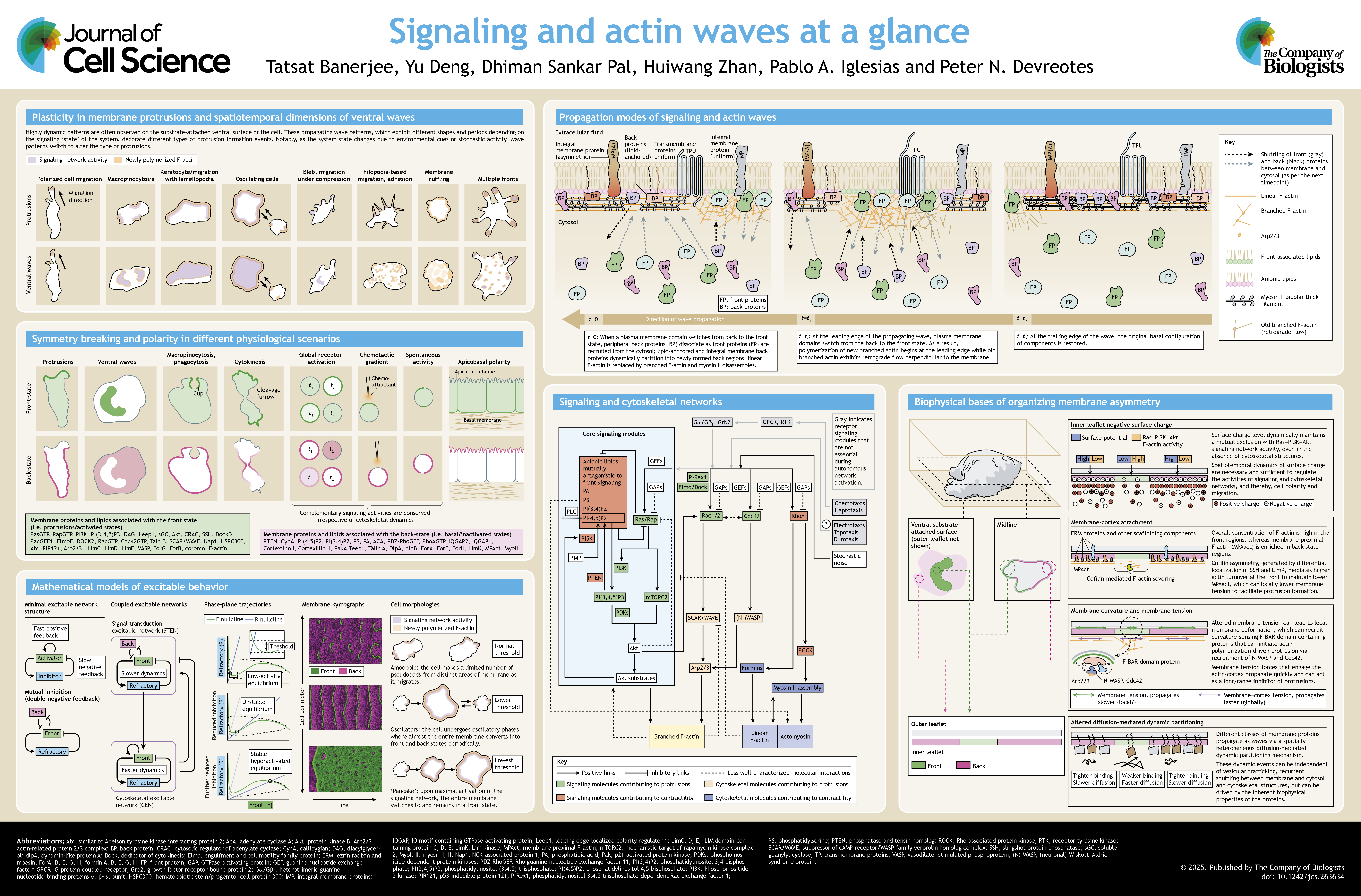

Supplement: Poster [file joces-138-263634-s1.jpg]

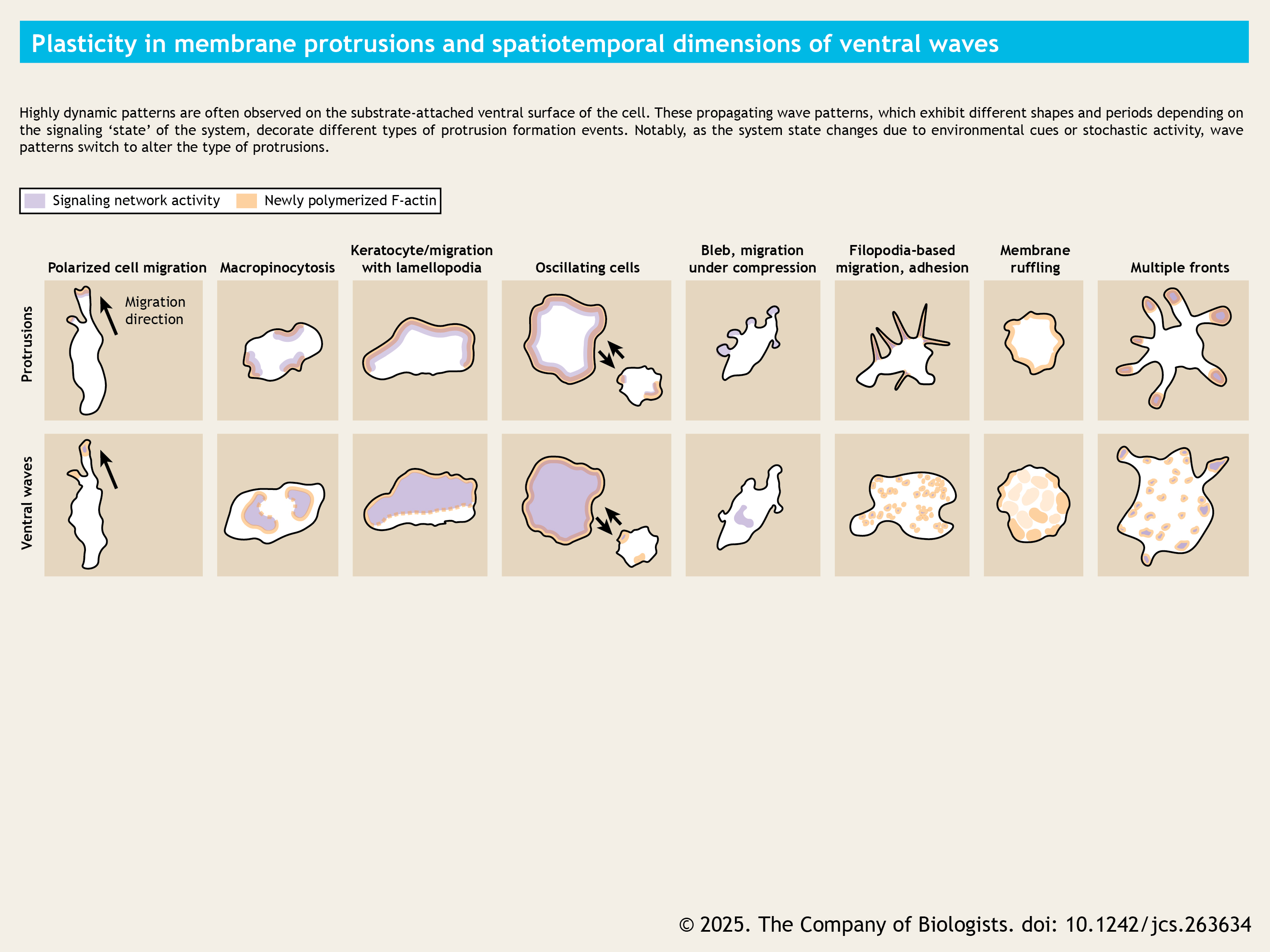

Supplement: Panel 1. Plasticity in membrane protrusions and spatiotemporal dimensions of ventral waves [file joces-138-263634-s2.jpg]

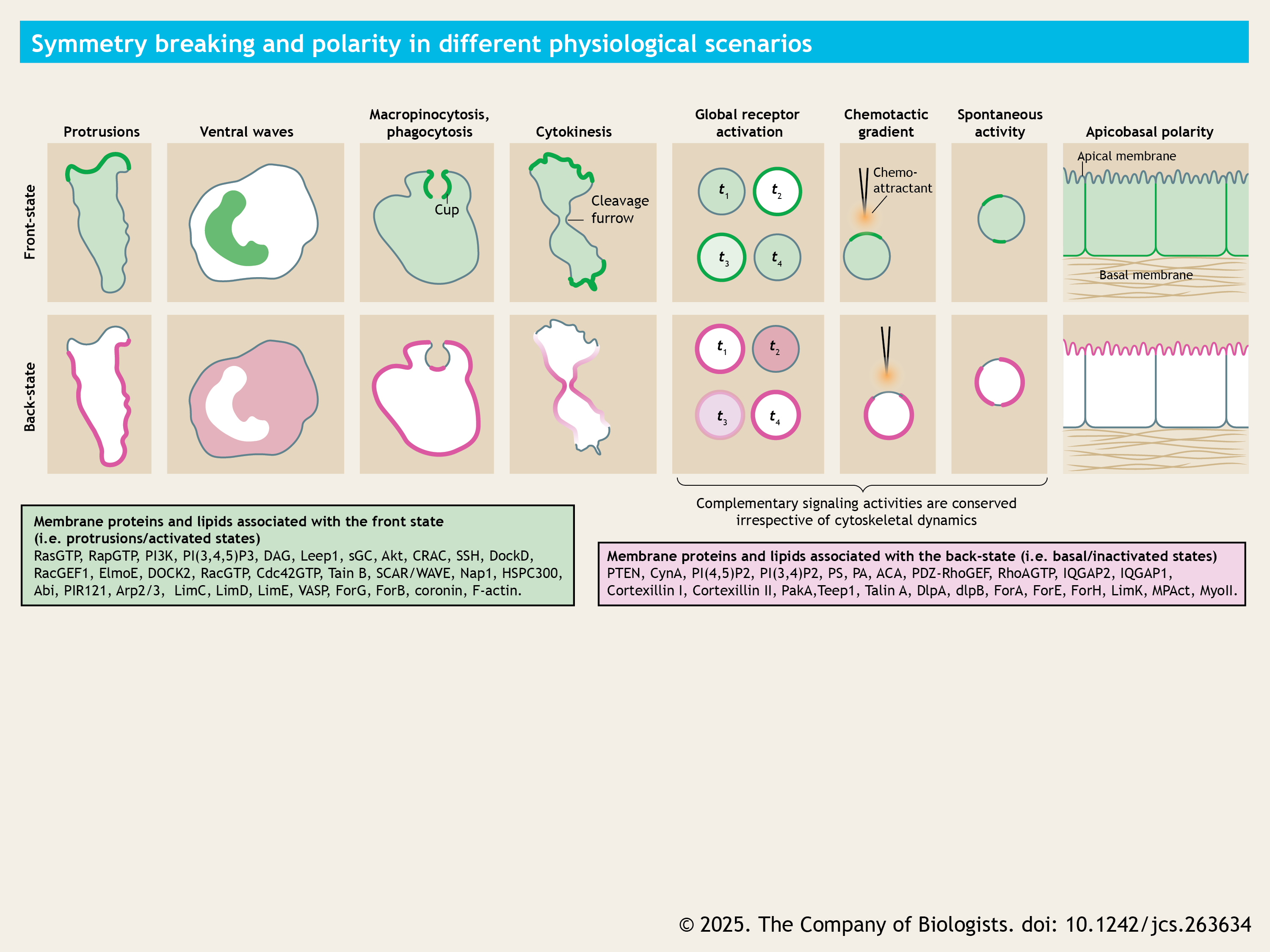

Supplement: Panel 2. Symmetry breaking and polarity in different physiological scenarios [file joces-138-263634-s3.jpg]

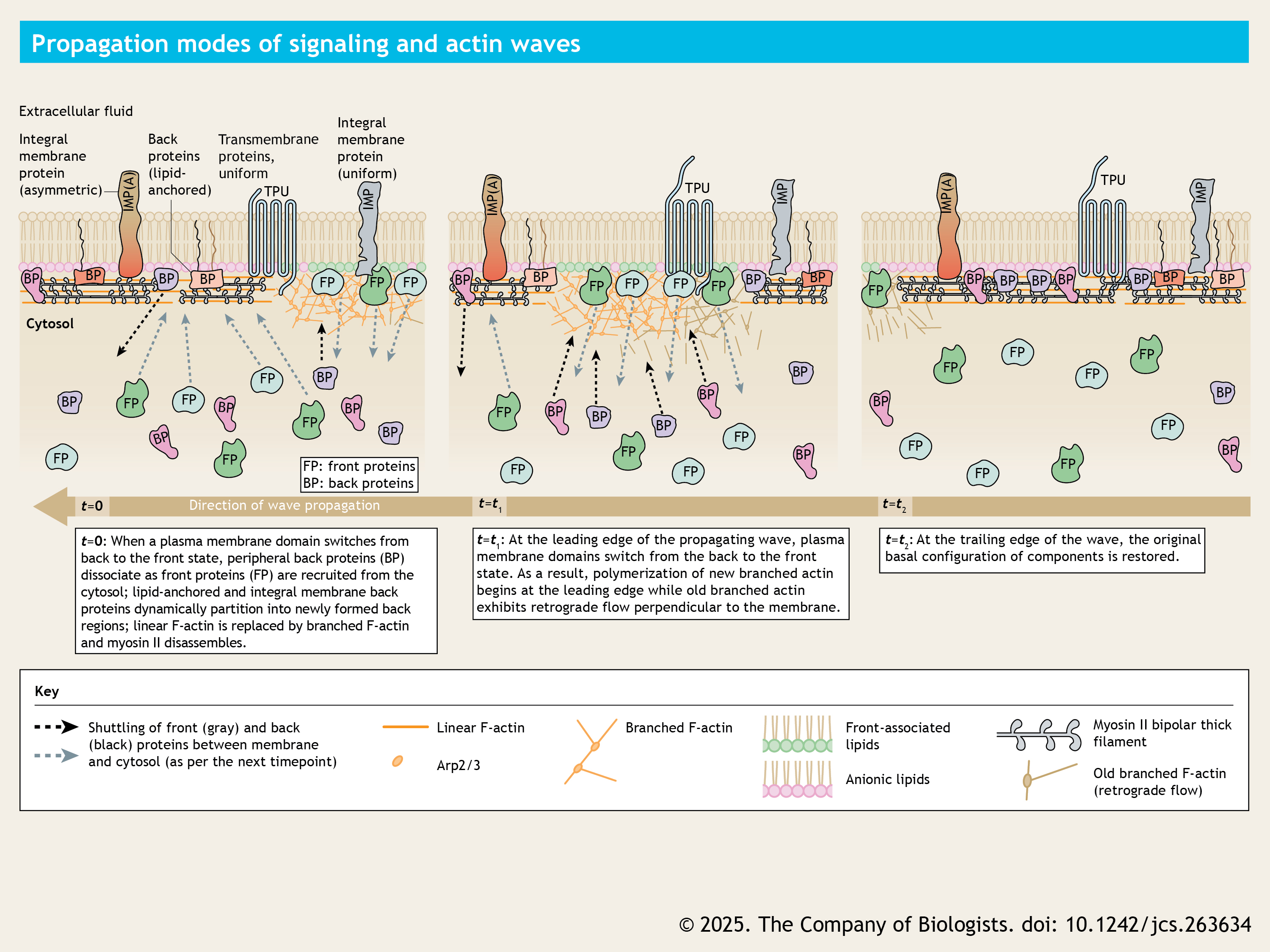

Supplement: Panel 3. Propagation modes of signaling and actin waves [file joces-138-263634-s4.jpg]

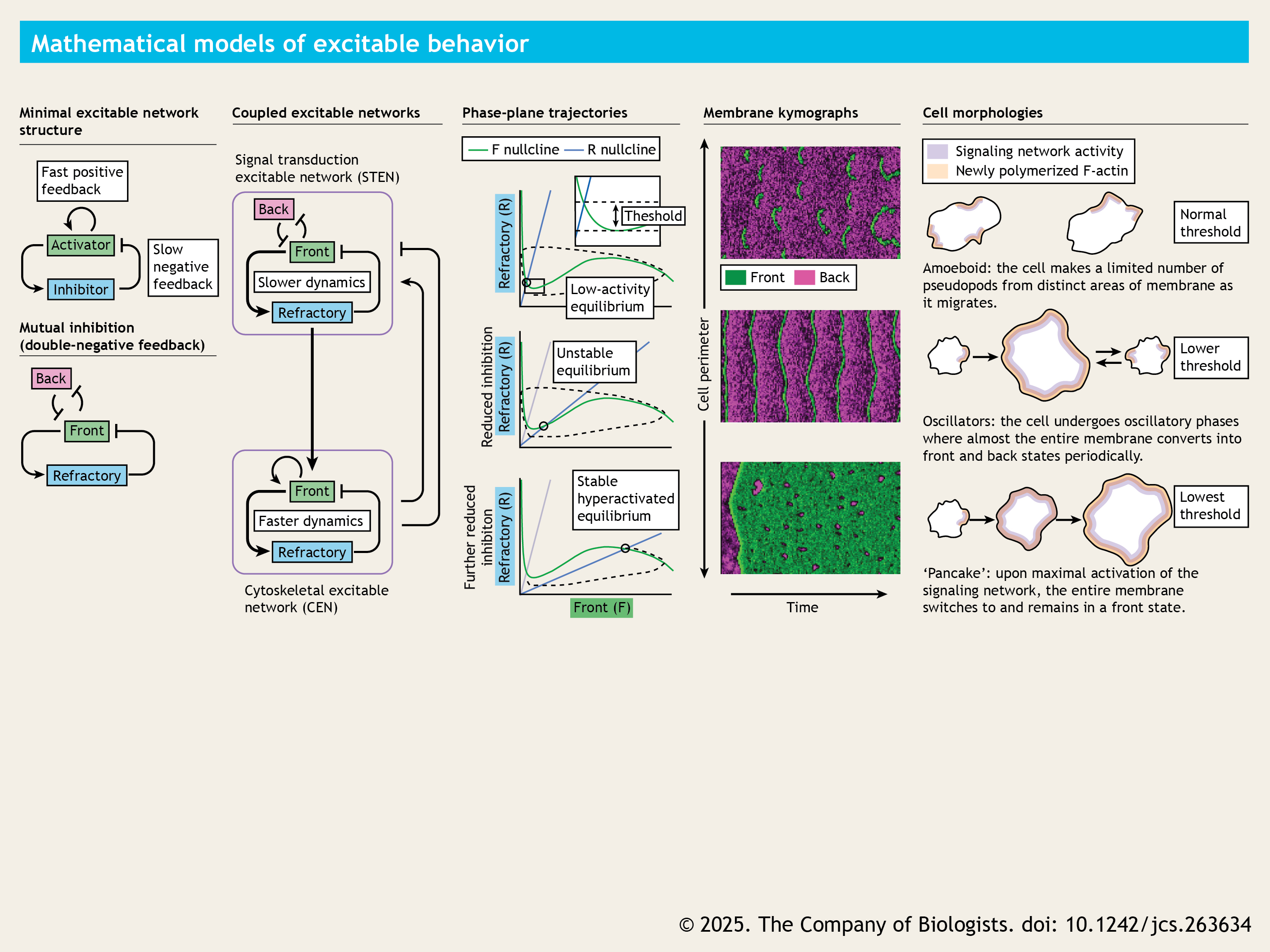

Supplement: Panel 4. Mathematical models of excitable behavior [file joces-138-263634-s5.jpg]

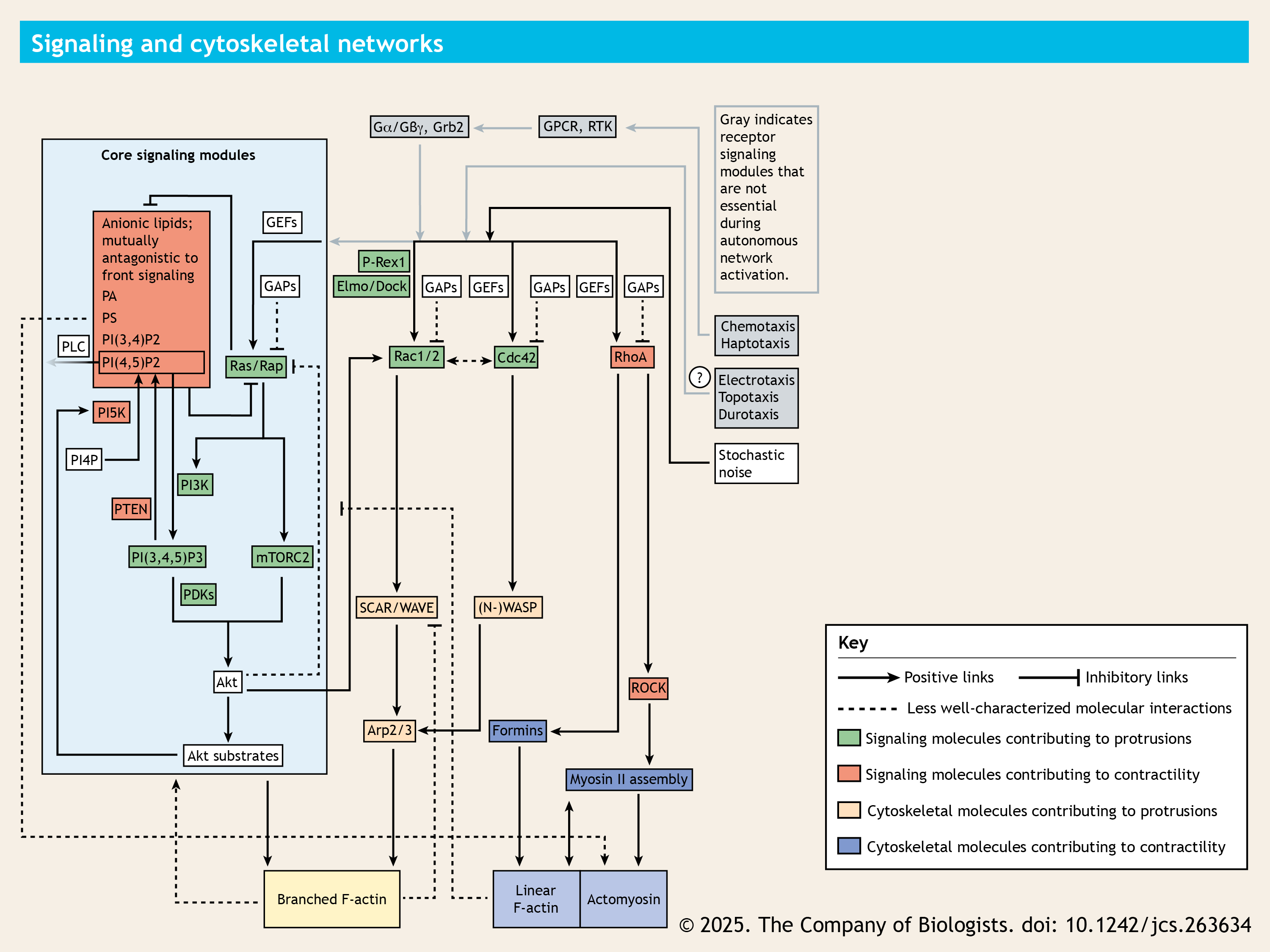

Supplement: Panel 5. Signaling and cytoskeletal networks [file joces-138-263634-s6.jpg]

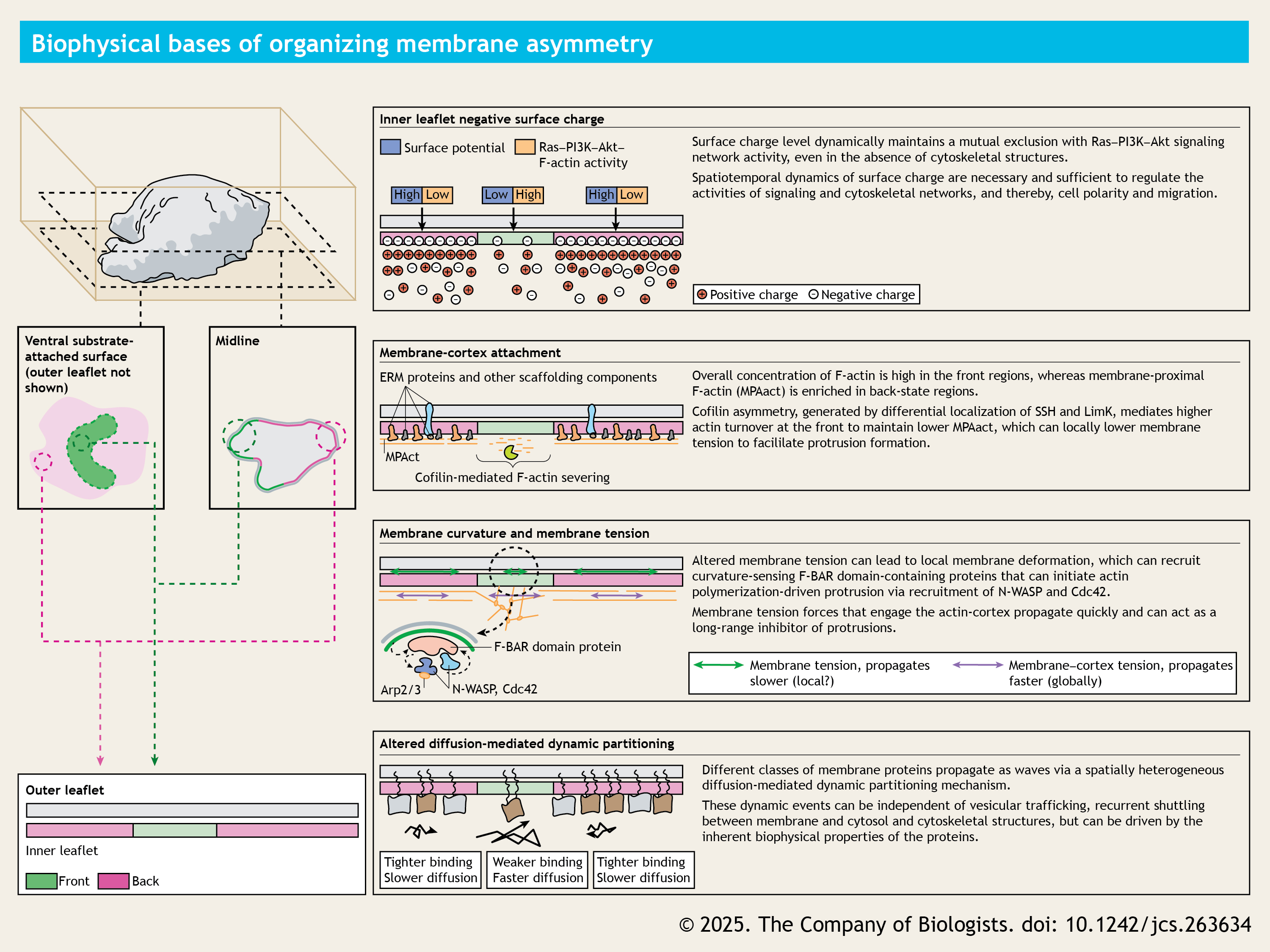

Supplement: Panel 6. Biophysical bases of organizing membrane asymmetry [file joces-138-263634-s7.jpg]
